# Comparing Machine Learning Models and Statistical Models for Predicting Heart Failure Events: A Systematic Review and Meta-Analysis

**DOI:** 10.3389/fcvm.2022.812276

**Published:** 2022-04-06

**Authors:** Zhoujian Sun, Wei Dong, Hanrui Shi, Hong Ma, Lechao Cheng, Zhengxing Huang

**Affiliations:** ^1^Zhejiang Lab, Hangzhou, China; ^2^Zhejiang University, Hangzhou, China; ^3^Department of Cardiology, The First Medical Center of Chinese PLA General Hospital, Beijing, China; ^4^Department of Cardiology, The Second Affiliated Hospital of School of Medicine, Zhejiang University, Hangzhou, China

**Keywords:** heart failure, prediction model, machine learning, systematic review, statistical model

## Abstract

**Objective:**

To compare the performance, clinical feasibility, and reliability of statistical and machine learning (ML) models in predicting heart failure (HF) events.

**Background:**

Although ML models have been proposed to revolutionize medicine, their promise in predicting HF events has not been investigated in detail.

**Methods:**

A systematic search was performed on Medline, Web of Science, and IEEE Xplore for studies published between January 1, 2011 to July 14, 2021 that developed or validated at least one statistical or ML model that could predict all-cause mortality or all-cause readmission of HF patients. Prediction Model Risk of Bias Assessment Tool was used to assess the risk of bias, and random effect model was used to evaluate the pooled c-statistics of included models.

**Result:**

Two-hundred and two statistical model studies and 78 ML model studies were included from the retrieved papers. The pooled c-index of statistical models in predicting all-cause mortality, ML models in predicting all-cause mortality, statistical models in predicting all-cause readmission, ML models in predicting all-cause readmission were 0.733 (95% confidence interval 0.724–0.742), 0.777 (0.752–0.803), 0.678 (0.651–0.706), and 0.660 (0.633–0.686), respectively, indicating that ML models did not show consistent superiority compared to statistical models. The head-to-head comparison revealed similar results. Meanwhile, the immoderate use of predictors limited the feasibility of ML models. The risk of bias analysis indicated that ML models' technical pitfalls were more serious than statistical models'. Furthermore, the efficacy of ML models among different HF subgroups is still unclear.

**Conclusions:**

ML models did not achieve a significant advantage in predicting events, and their clinical feasibility and reliability were worse.

## Introduction

Heart failure (HF), as a complex cardiovascular syndrome, causes severe healthcare burdens, and its prevalence continues to increase with the global aging tendency (1). Despite recent improvements in diagnosis and management, HF prognosis remains poor (1, 2), partly because estimating patient risk is difficult (3, 4). Due to this challenge, prediction models are considered a potential tool to help clinicians make informed decisions about treatment initiation and survival estimation to prevent adverse HF events (5).

In recent years, machine learning (ML) models have been suggested to be a revolutionary innovation with the potential to transform the whole healthcare system (6) and have been gradually leveraged to create prognostic prediction models. Concerning this changing trend, existing HF prediction models can be coarsely divided into two categories based on methodologies: statistical and ML models. Although ML models are typically described to have theoretical superiority over statistical models for their ability to fit complex data patterns (7), previous studies arrived at controversial conclusions. Some studies claimed that ML-based methods were indeed better than statistical models (8–10), while others held opposite views (11–13). The conflicting opinions on the superiority of ML models motivated us to review HF prediction models with respect to their methodologies comprehensively to answer two questions. (1) Does ML models obtained better performance in predicting HF events? (2) What are the weaknesses of current ML models?

Of note, previous reviews on HF prediction models generally only focused on their discrimination ability (14–19), while the other factors that may affect model application were usually ignored. In this review, we analyzed two representative prognostic events in HF, all-cause death and all-cause readmission. We compared the c-index of the two types of models and investigated their reliability and clinical feasibility, which may clarify the current position of ML models in the two HF events prediction research and identify directions for future work.

## Methods

We conducted this analysis according to the Preferred Reporting Items for Systematic Reviews and Meta-Analysis (PRISMA) statement (20) and the Checklist for Critical Appraisal and Data Extraction for Systematic Reviews of Prediction Modeling Studies (CHARMS) (21).

Of note, there is no clear demarcation between the two types of models (7). In this review, statistical models refer to linear models developed by logistic regression or Cox regression or those presented as risk scoring systems. ML models refer to emerging models developed by ML methods only.

### Literature Search

We designed a broad literature search strategy to include all articles published in English between 2010 and 2021 by applying a search string to their title, abstract, or keyword sections. Earlier studies were not included due to possible discrepancies in population characteristics and therapy. The search string was “(predict^*^ OR progn^*^ OR “risk assessment” OR “risk calculation”) AND “heart failure” AND (model OR algorithm OR score).” ZJS searched Medline, Web of Science, and IEEE Xplore on July 14, 2021, and included 13,301 papers for further analysis.

### Selection Procedure

We analyzed two representative prognostic events in HF, all-cause death and all-cause readmission. Articles that reported the development or validation of at least one statistical or ML model for predicting these events with appropriate performance evaluation were included. Models that predicted other outcomes or composite endpoints were excluded. Appropriate performance evaluation indicated that each model should report the c-index in the validation phase. Studies that only reported the c-index of models on the training dataset were excluded. Studies also need to report the 95% confidence interval (CI) of the c-index as well [or sample size and event number such that the 95% CI of the c-index could be calculated approximately (22)]. We excluded models that were not originally designed to predict HF events (e.g., CHA2DS2-VASC) (23). These models may be transferred to predict HF events to highlight associations between other disease processes and HF prognosis.

We conducted a three-phase selection in practice as we have more than 10 thousand papers to review. First, we only reviewed papers via their titles, and the papers whose topic is not predicting the two representative prognostic HF events will be excluded. Then, we reviewed papers according to their abstracts, and the papers which did not report any quantitative metrics will be excluded. Finally, we conducted the full-text review, and only the papers that meet all requirements described above will be reserved. If we cannot identify whether a paper should be included via its title/abstract, we will reserve the paper into the next phase for a more detailed investigation. ZS, HS independently conducted the selection procedure, and the discrepancy was reviewed by HM.

### Data Extraction

Three researchers (ZH, WD, and HM) performed data extraction by following recommendations in the CHARMS statement. From all eligible articles, two researchers (ZS, HS) extracted (as applicable) the first author, title, digital object identifier, journal, geographical location, year of publication, study type, model name, data collection manner, patient selection criteria, predictor selection method, missing data processing method, numerical feature processing method, age, gender ratio, sample size, number of events, predicted outcome, follow-up time, c-index, 95% CI of c-index, type of algorithm, and performance validation method. HF subtype was also extracted to take HF heterogeneity into consideration. We also collected the list of predictors to investigate their usage.

### Statistical Analysis

We summarized the basic characteristics of the studies with respect to the type of methods and prediction tasks. The summarized characteristics included algorithm, geographical location, admission type (chronic or acute HF), HF type (HF with reduced ejection fraction, HFrEF, or HF with preserved ejection fraction, HFpEF), publication year, and study type (model development study or validation study). The performances of statistical and ML models were compared from two perspectives: (1) We conducted random effects model based meta-analysis to compare the pooled c-index of the two methodologies for all included studies. The meta-analysis was conducted via MedCalc (24), and I-square is used to evaluate the heterogeneity of meta-analysis. (2) We conducted a “head-to-head” performance comparison for studies that developed both ML models and statistical models, which helped us to explore the performance gain of utilizing ML methods under the same experimental settings.

### Risk of Bias Assessment

Three researchers (ZH, WD, and ZH) adopted the Prediction Model Risk of Bias Assessment Tool (PROBAST) to appraise reliability (25). PROBAST evaluates the risk of bias (ROB) of models in four domains: participants, predictors, outcome, and statistical analysis. Each domain contained several ROB signal questions answered with “yes,” “probably yes,” “no,” “probably no,” or “no information,” and the domains were ranked independently. If answers to all questions in all four domains were “yes” or “probably yes,” the model was regarded as “low ROB”; if “yes” to all questions in the four domains, the model was with “high ROB.” If a domain contained at least one question that signaled “no information,” and no question was answered “no” or “probably no,” the domain was graded “unclear ROB.” If at least one domain was regarded as unclear and none as high, the model was also graded “unclear ROB.”

## Results

### Characteristics of Models

**Figure 1:**
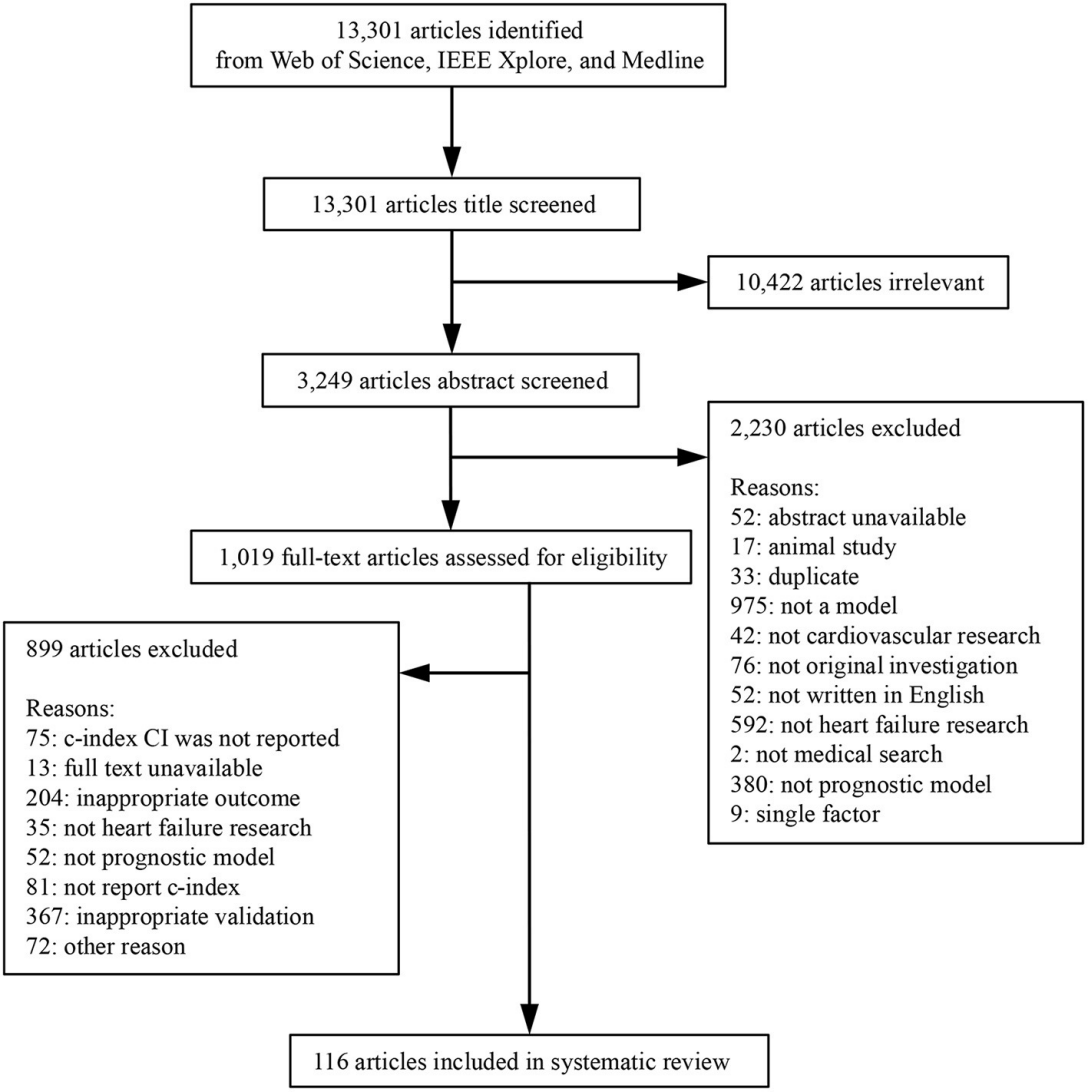
Literature selection procedure.

After screening (Figure 1), 280 models from 116 articles were selected, the details of which are shown in Supplementary Table 1, Supplementary Data Sheet. Table 1 describes the basic characteristics of the included models, the excluded articles and corresponding reasons were included in the Supplementary Material as well. Among all 280 model studies, 68% of the statistical model studies and 95% of the ML model studies were conducted in the last 5 years (2016–2021). The included studies were mainly from North America (41%), Europe (31%), and East Asia (22%). Concerning the outcomes, 205 (73%) models predicted all-cause mortality, and 75 (27%) predicted all-cause readmission. Among the 202 statistical model studies, 101 (50%) studies were model development studies, and 101 (50%) were validation studies of 31 different statistical models, while all ML model studies were model development studies. ML models were grouped into seven types according to the algorithm, where boosting, random forest, and decision tree were the most used methods.

**Table 1:** Model characteristics.

	**Overall**	**Statistical model**		**Machine learning model**			
	**280 models**	**Overall 202 models**	**Mortality 158 models**	**Readmission 44 models**	**Overall 78 models**	**Mortality 47 models**	**Readmission 31 models**
**HF type**							
Acute HF	73 (26%)	66 (33%)	62 (39%)	4 (9%)	7 (9%)	6 (13%)	1 (3%)
Chronic HF	32 (11%)	32 (16%)	25 (16%)	7 (16%)	0 (0%)	0 (0%)	0 (0%)
Not specified	175 (62%)	104 (51%)	71 (44%)	33 (75%)	71 (91%)	41 (87%)	30 (97%)
**LVEF**							
HFrEF	65 (23%)	62 (31%)	55 (35%)	7 (16%)	3 (4%)	3 (6%)	0 (0%)
HFpEF	23 (8%)	13 (6%)	11 (7%)	2 (5%)	10 (13%)	10 (21%)	0 (0%)
Not specified	192 (69%)	127 (63%)	92 (58%)	35 (80%)	65 (83%)	34 (72%)	31 (100%)
**Admission type**							
Inpatient	172 (61%)	127 (63)	98 (62%)	29 (66%)	45 (58%)	25 (53%)	20 (65%)
Outpatient	45 (16%)	37 (18%)	32 (20%)	5 (11%)	8 (10%)	8 (17%)	0 (0%)
Other ^*^	63 (22%)	38 (19%)	28 (18%)	10 (23%)	25 (32%)	14 (30%)	11 (35%)
**Region**							
North America	116 (41%)	72 (36%)	47 (30%)	25 (57%)	44 (56%)	27 (57%)	17 (55%)
Europe	88 (31%)	78 (39%)	67 (42%)	11 (25%)	10 (13%)	2 (4%)	8 (26%)
East Asia	61 (22%)	40 (20%)	35 (22%)	5 (11%)	21 (27%)	18 (38%)	3 (10%)
Others	15 (5%)	12 (6%)	9 (6%)	3 (7%)	3 (4%)	0 (0%)	3 (10%)
**Algorithm**							
Cox regression	64 (23%)	64 (32%)	58 (37%)	6 (14%)	/	/	/
LR	61 (22%)	61 (30%)	31 (20%)	30 (68%)	/	/	/
Score	77 (28%)	77 (38%)	69 (44%)	8 (18%)	/	/	/
RF	11 (4%)	/	/	/	11 (14%)	7 (15%)	4 (13%)
Boosting	17 (6%)	/	/	/	17 (22%)	11 (23%)	6 (19%)
SVM	7 (3%)	/	/	/	7 (9%)	5 (11%)	2 (6%)
Neural network ^**^		/	/	/			
Multi-layer perceptron	7 (3%)	/	/	/	7 (9%)	5 (11%)	2 (6%)
Deep learning	8 (3%)	/	/	/	8 (10%)	2 (4%)	6 (19%)
Decision tree	10 (4%)	/	/	/	10 (13%)	8 (17%)	2 (6%)
Others	18 (6%)	/	/	/	18 (23%)	9 (19%)	9 (29%)
**Year of publication**							
2010–2015	69 (25%)	65 (32%)	50 (32%)	15 (34%)	4 (5%)	2 (4%)	2 (6%)
2016–2021	211 (75%)	137 (68%)	108 (68%)	29 (66%)	74 (95%)	45 (96%)	29 (94%)
**Study type**							
Model development	179 (64%)	101 (50%)	71 (45%)	30 (68%)	78 (100%)	47 (100%)	31 (100%)
Model validation	101 (36%)	101 (50%)	87 (55%)	14 (32%)	0 (0%)	0 (0%)	0 (0%)

*Values are presented as numbers*.

* *“Others” indicates that studies did not specify the origin of patients, or patients have mixed origins*.

** *The deep learning model refers to recently proposed neural network-based models (e.g., recurrent neural nets and autoencoder) apart from simple multi-layer perceptron*.

*HF, heart failure; HFpEF, heart failure with preserved ejection fraction;HFrEF, heart failure with reduced ejection fraction; LVEF. left ventricular ejection fraction*.

Table 1 also indicates the efficacy of statistical HF models was widely investigated for different patient subgroups, as 33% of statistical models were developed or validated for acute HF patients, 16% for chronic HF patients, 31% for HFrEF patients, 6% for HFpEF patients, 63% for hospitalized patients, and 18% for ambulatory patients. In comparison, most ML models did not consider the heterogeneity of HF. ML models were typically developed using a “general” HF population dataset that identified HF patients by primary diagnoses or international classification of disease (ICD) codes without other inclusion or exclusion criteria. For example, these studies did not take symptoms, left ventricular ejection fraction (LVEF), admission type, and comorbidities into consideration to select a specific HF patient group.

Concerning the predictor usage characteristic, statistical models used significantly fewer predictors than ML models (Supplementary Figure 1). Specifically, 93 out of 101 (92%) statistical model development studies reported the number of predictors with the median was 11 (interquartile range, IQR: 6–18), and 76% of studies used less than 20 predictors. All ML model development studies reported the number of predictors; the median was 62 (IQR: 16–516), and 72% of studies used more than 20 predictors. Sixty-six (67%) statistical models reported a detailed list of predictors, while only 36 (46%) ML model development studies reported a detailed list of predictors. The predictor usage details can be found in Supplementary Figure 2.

### Performance Comparison

**Figure 2:**
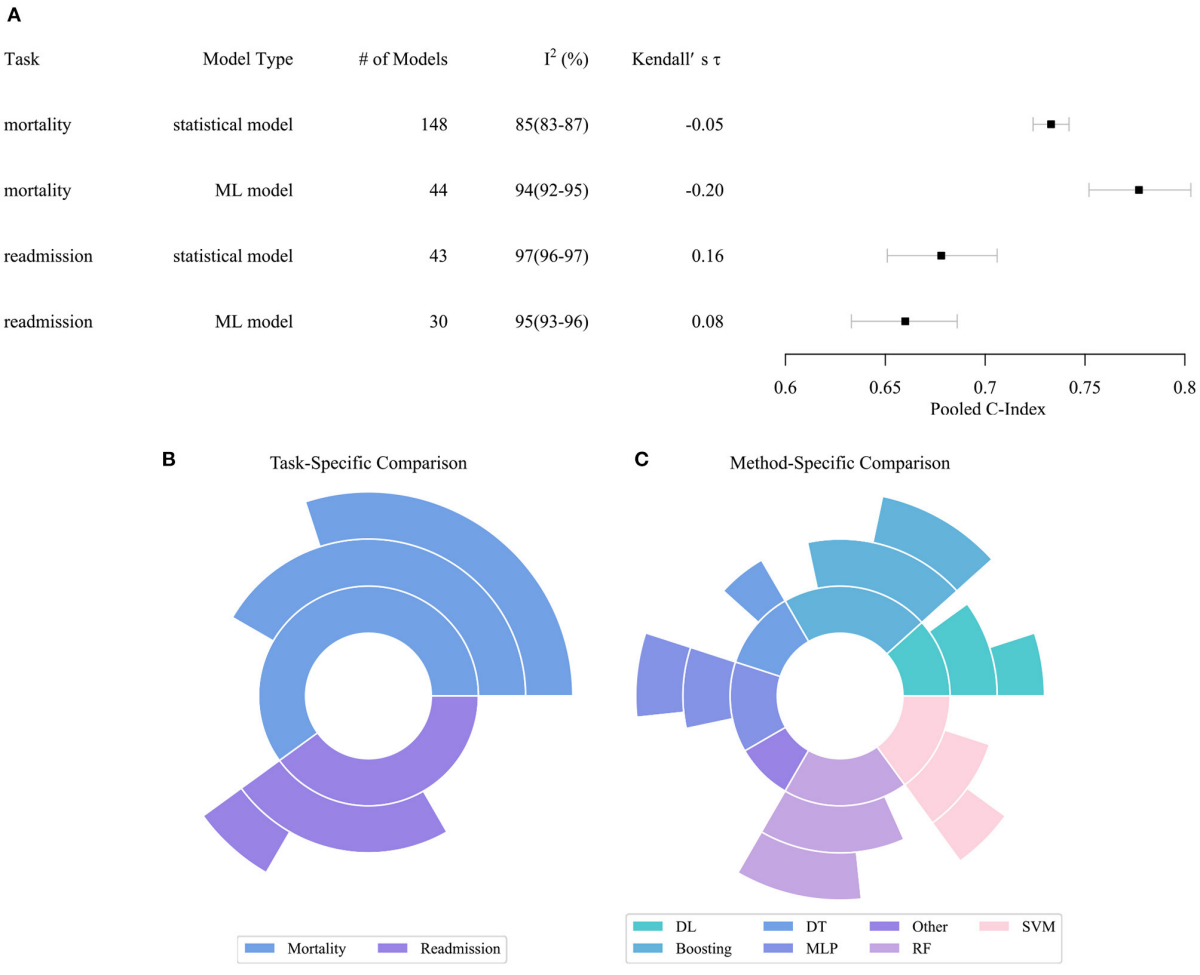
Performance distribution of statistical and ML models. **(A)** Pooled C-index of meta-analysis with respect to tasks and model types. **(B)** Task specific comparison result. **(C)** Model specific comparison result. In subplots **(B,C)**, the inner pie indicates the number of pairwise comparisons between ML models and statistical models. The middle pie indicates the number of pairs in which the ML model achieved better performance, while the outer pie indicates the number of pairs in which the superiority of ML models reached statistical significance. ML, Machined Learning; DL, Deep Learning; DT, Decision Tree; SVM, Support Vector Machine; MLP, Multi-Layer Perceptron; RF, Random Forest.

Figure 2 describes the performance comparison result of the models. As shown in Figure 2A, the pooled c-index of statistical models in predicting all-cause mortality, ML models in predicting all-cause mortality, statistical models in predicting all-cause readmission, ML models in predicting all-cause readmission were 0.733 (95% CI 0.724–0.742), 0.777 (0.752–0.803), 0.678 (0.651–0.706), and 0.660 (0.633–0.686), respectively. The meta-analysis indicated that the ML model only outperformed the statistical model in predicting all-cause mortality. In contrast, their performance in predicting all-cause readmission was worse than the statistical model. Of note, the predictive ability across all studies exhibited substantial heterogeneity. Even when these characteristics were incorporated in the meta-regression model or multi-level meta-analysis model, heterogeneity remained high and essentially unchanged. Since the substantial heterogeneity of the included models did not permit reliable comparison of performance by meta-analysis, the result of meta-analysis can only be interpreted carefully within the context.

Figures 2B,C describe the result of the head-to-head comparison, which compares the performance differences of ML models and statistical models trained by same dataset. We found 60 valid comparison pairs, of which ML models achieved better performance in 39 (65%) pairs, and the superiority reached statistical significance in 22 (37%) pairs. In the task-specific comparison, we observed that ML models had a substantial advantage in predicting all-cause mortality, which accords to the result of the meta-analysis. ML models obtained better performance in two-thirds of the pairs, and the advantage was statistically significant in half of the pairs. ML models achieved similar performance compared to statistical models in predicting all-cause readmission. In the method-specific comparison, deep neural networks, boosting, multi-layer perceptron, support vector machine (SVM), and random forest (RF) were more likely to achieve better performance than statistical models, while decision tree and other ML algorithms achieved comparable or even worse performance.

### Risk of Bias Assessment

**Figure 3:**
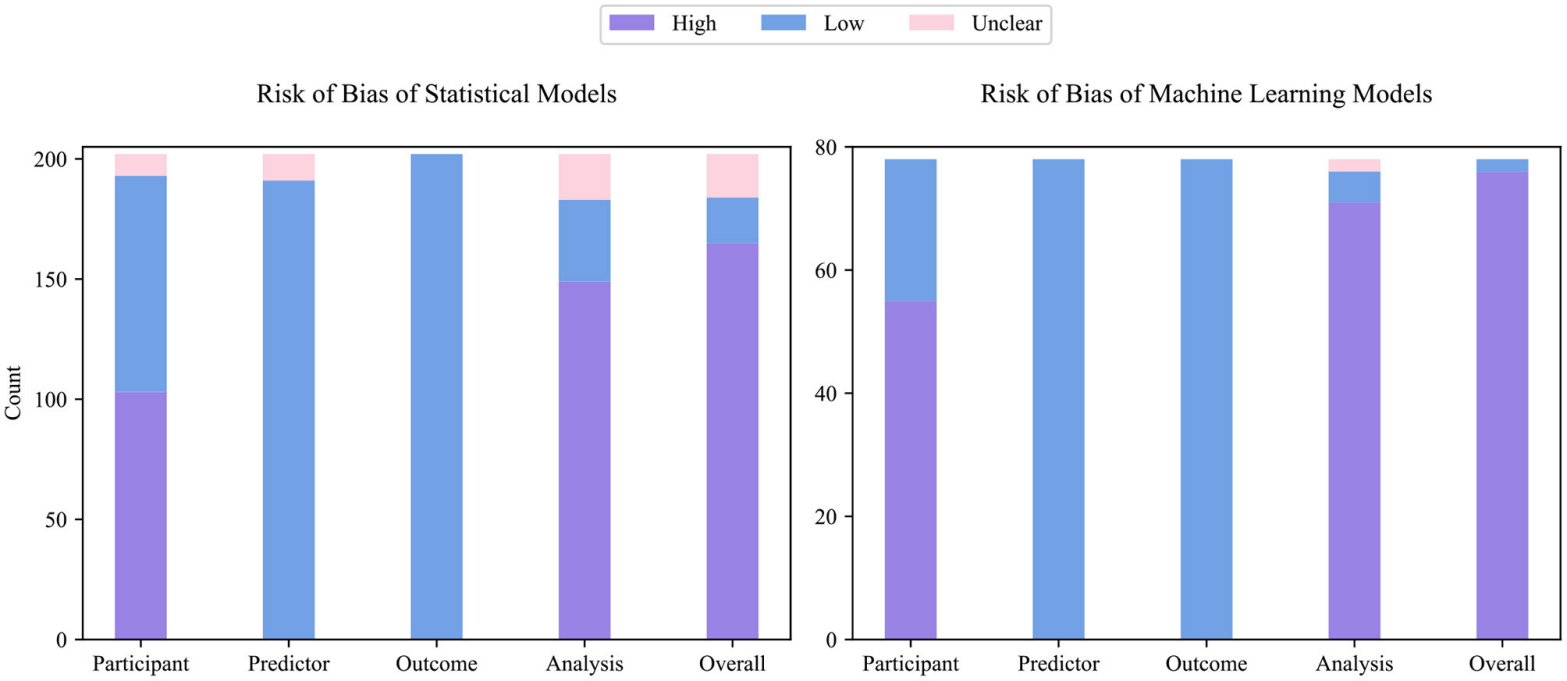
Result of risk of bias analysis.

Figure 3 describes the ROB of both statistical and ML models. Among 202 statistical models and 78 ML models, only 19 statistical and 2 ML models were graded as low ROB, 18 statistical models were graded as unclear ROB, while all remaining models were graded as high ROB, indicating that most prediction models have technical pitfalls. The measured ROB was mainly from the participant and analysis domains. In PROBAST, models derived from retrospective data were treated as high participants ROB, and 103 (51%) statistical and 55 (70%) ML models were developed or validated with a retrospective dataset. One hundred and sixty-five (82%) statistical and 76 (97%) ML model studies did not conduct appropriate statistical analysis or reported incomplete statistical analysis information. Specifically, 22 (11%) statistical and 54 (69%) ML models were developed or validated from an insufficient number of participants. Thirty-seven (36%) statistical and 21 (27%) ML models categorized continuous predictors, which caused unnecessary loss of information (25). While 52 (26%) statistical and 24 (31%) ML models used high-risk imputation methods, 65 (32%) statistical and 35 (45%) ML models did not report data imputation in detail. To select predictors, 31 (31%) statistical and 9 (12%) ML models used univariate analysis. Although the univariate analysis is a widely adopted method, its use has been discouraged recently (26). One hundred and three (51%) statistical and 62 (80%) ML models did not conduct (or conducted inappropriate) calibration evaluation. Thirty-five (35%) statistical and 34 (44%) ML models used random split or non-random split, rather than more reliable bootstrap and cross-validation, to evaluate the discrimination ability of models.

## Discussion

### Discrimination Ability

Different from the conclusion of previous reviews (17), our analysis indicated that the performance of ML models is not consistently better than statistical models in predicting HF prognostic events. The superiority of ML models significantly relies on specific experimental environments, i.e., prediction events, population characteristics, and selected algorithms. The meta-analysis result indicated ML models obtained better performance in predicting all-cause mortality, while their performance was worse in predicting all-cause readmission. The head-to-head analysis indicated that ML models achieved similar or worse performance in about one-third of pairwise cases even in exactly the same experimental settings. For the cases where ML models achieved better performance, their superiority is probably not statistically significant. We also found not all ML algorithms were superior to statistical models. Specifically, ensemble learning-based models (boosting and RF) and neural network-based models (multi-layer perceptron and deep learning) achieved better performance, while decision tree and support vector machines generally achieved worse performance than statistical models. This finding is in accordance with the consensus in the computer science community (27, 28).

According to these results, we argue that the expectation of leveraging ML has not yet been fulfilled. ML models were suggested to transfer their computer vision and natural language processing success, thereby transforming medicine (6). Although we cannot precisely summarize the performance of statistical and ML models in event prediction due to their heterogeneity, ML models clearly achieved at most moderately better or comparable performance compared to statistical models. This degree of improvement is unlikely to trigger a revolution in HF event prediction.

However, the potential of ML models still warrants further investigation for two reasons. First, current ML studies did not take full advantage of the data being analyzed. ML models typically require a large training dataset to become efficient and avoid overfitting. However, our ROB analysis showed about three-quarters of ML models were developed using insufficient numbers of participants, as their event per variable rate was less than 10 (29). ML models were also more likely to be trained by low-quality electronic health record datasets, which also negatively affected their performance. Meanwhile, as HF is a chronic syndrome, longitudinal clinical patient data usually accumulate, including treatment, laboratory tests, and image information generated over the entire HF management process. It is worth investigating how to extract information from longitudinal sequential datasets to achieve better event prediction performance, and only ML algorithms are capable of handling this task (30). Second, the performance of ML models was not sufficiently validated. Notably, about half of the statistical model studies were validation studies, whereas no ML models were externally validated. As model performance typically degenerates upon validation, the performance of current ML models is probably overestimated, but to what degree is unclear.

### Model Reliability

This review revealed two reliability issues of ML models, the first being neglect of HF heterogeneity. As a complex syndrome, HF prognosis varies widely among different patient subgroups (19). We observed statistical model studies generally identified this situation and applied a detailed sample inclusion procedure to select the target population before developing or validating a model. Therefore, the efficacy of statistical models in each HF subgroup, e.g., HFrEF, HFpEF, acute HF, and chronic HF, has already been investigated. On the contrary, ML models were typically developed using a “general” HF patient group without clear inclusion criteria and only reported overall performance metrics. The subgroup-specific performance of ML models is currently unclear, which undoubtedly influenced the reliability of ML models in our study. The efficacy of ML models in different HF patient subgroups needs to be investigated in future studies.

Secondly, the PROBAST analysis indicated both types of models have technical flaws. The issues affecting ML models were more serious and can be coarsely summarized to four points. Insufficient information disclosure was the first flaw. PROBAST analysis demonstrated that most statistical and ML models did not report sufficient statistical analysis information, and no ML models reported enough details for model reproduction. Inappropriate statistical analysis was the second flaw. ML models systematically did not perform calibration analysis, which may lead to inaccurate evaluation of event risk. Meanwhile, the training dataset of a large fraction of ML studies was too small to optimize parameters satisfactorily. Third, it is worth noting that adopting ML methods and proposing low ROB models were sometimes controversial. For example, as ML models usually require a large dataset for training, the time cost of imputing missing data via multiple imputation algorithms usually becomes intolerable. In fact, ML studies typically use mean-value imputation or a separate category to tackle the missing data problem, which inevitably brings ROB to ML models and makes them untrustworthy. Lack of external validation was the fourth flaw. More than half of statistical model studies were validation studies, while no ML model was externally validated by independent studies. ROB analyses indicated that the lack of a practical guideline in ML model development and validation is a big challenge. Such a guideline could help tackle a series of tasks in ML model development (i.e., data collection, pre-processing, performance evaluation, and model releasing), and thereafter provide a feasible path for developing reliable ML models, rather than just adopting a particular ML algorithm to fit a clinical dataset.

### Clinical Feasibility

Our analysis indicated that the clinical feasibility of ML models was low due to immoderate usage of predictors and lack of computer infrastructure, which may explain why no ML model was externally validated. Current ML development studies usually waived the predictor selection process, which is essential to developing statistical models. As a result, ML models included on average eight times the number of predictors used in statistical models; some even used more than 1,000 predictors. Although the inclusion of more information was regarded as an advantage of ML-based models (31), it makes the model prone to overfit. Furthermore, with more predictors to be evaluated, the inclusion of a large number may limit the clinical utility of models. ML models require a feature selection protocol to effectively utilize more patient information in order to make a precise prediction and determine clinical feasibility.

The complexity of the ML algorithm and the number of used predictors indicated that it is impossible to calculate the model manually. A comprehensive pipeline, including data collection, pre-processing, model invocation, and results display needed to execute a model in real-time. The development of the pipeline requires mass interdisciplinary collaboration, and the pipeline needs to be elaborately integrated into the current clinical decision support system. As the development and deployment of such a pipeline are obviously beyond the ability of most hospitals, ML models are difficult to be applied in clinical practice. An updated medical decision-making system is required to facilitate the deployment of ML models in the clinical workflow.

### Strengths and Limitations of This Study

The major strength of this study was that we analyzed the clinical feasibility and reliability of statistical and ML models, which were not systematically investigated in the previous review (14–19). The large number of studies used to compare different ML and statistical models was another major strength. However, this study also had several limitations. (1) The heterogeneity among studies impeded us from conducting rigorous performance comparisons of the two types of models. (2) We did not conduct an analysis on the calibration of models, as most studies did not report calibration information or conducted inappropriate calibration. (3) We only analyzed for all-cause mortality events, due to the lack of relevant studies examining all-cause readmission. Findings in this review may not be generalizable to other outcomes or settings.

## Conclusion

In summary, our review indicated ML models did not show the ability to revolutionize the process of predicting prognosis, and due to lack of external validation, their performance is probably overestimated. It seems the increased complexity of ML models did not bring significantly better performance. However, we argue it is still too early to claim that introducing ML technique in heart failure event prediction task is as meaningless. Because applying ML technique in medicine is an emerging research area and pioneer studies may be immature. Substantial effort is required in the future to explore how to utilize ML technology to achieve precise event prediction and overcome the difficulties in deploying ML models in clinical environments.

## Data Availability Statement

The original contributions presented in the study are included in the article/Supplementary Material, further inquiries can be directed to the corresponding author.

## Author Contributions

ZS, WD, and ZH were involved in the conception and design of the review, developed the search strategy, and performed the study selection. ZS and HS extracted data from the included studies and were involved in the data analysis. ZS, WD, ZH, LC, and HM were involved in the interpretation and discussion of results. All authors drafted the manuscript, contributed to the drafting of the review, and revised it critically for important intellectual content.

## Funding

This work is being supported by Open Research Projects of Zhejiang Lab (No. 2019KD0AD01/012). The supporting bodies had no influence on the analysis and data, on the writing of the report, or on the decision to submit the paper for publication.

## Conflict of Interest

The authors declare that the research was conducted in the absence of any commercial or financial relationships that could be construed as a potential conflict of interest.

## Publisher's Note

All claims expressed in this article are solely those of the authors and do not necessarily represent those of their affiliated organizations, or those of the publisher, the editors and the reviewers. Any product that may be evaluated in this article, or claim that may be made by its manufacturer, is not guaranteed or endorsed by the publisher.

## References

[1] Dunlay SM Roger VL Redfield MM. Epidemiology of heart failure with preserved ejection fraction. Nature Rev Cardiol. (2017) 14: 591–602. 10.1038/nrcardio.2017.65 28492288

[2] Chioncel O Lainscak M Seferovic PM Anker SD Crespo-Leiro MG . Epidemiology and 1-year outcomes in patients with chronic heart failure and preserved, mid-range and reduced ejection fraction: an analysis of the ESC heart failure long-term registry. Eur J Heart Fail. (2017) 19:1574–574. 10.1002/ejhf.813 28386917

[3] Woldman S. Heart failure management-time to change our script on prognosis? Eur J Heart Fail. (2018) 20:837–8. 10.1002/ejhf.1157 29493084

[4] Banerjee P Watson C Ali D. Discussing prognosis in heart failure: a questionnaire-based study of the patient's view. JACC-Heart Failure. (2018) 6:803–4. 10.1016/j.jchf.2018.04.001 30166026

[5] Ponikowski P Voors AA Anker SD Bueno H Cleland JGF Coats AJS . 2016 ESC Guidelines for the diagnosis and treatment of acute and chronic heart failure. Eur Heart J. (2016) 37:2129–U130. 10.1093/eurheartj/ehw128 27206819

[6] Beam AL Kohane IS. Big data and machine learning in health care. JAMA. (2018) 319:1317–8. 10.1001/jama.2017.18391 29532063

[7] Rajkomar A Dean J Kohane I. Machine learning in medicine. New Eng J Med. (2019) 380:1347–58. 10.1056/NEJMra1814259 30943338

[8] Mudunuru VR Skrzypek LA A A comparison of artificial neural network and decision trees with logistic regression as classification models for breast cancer survival. Int J Math Eng Manag. (2020) 5:1170–90. 10.33889/IJMEMS.2020.5.6.089

[9] Benedetto U Dimagli A Sinha S Cocomello L Gibbison B Caputo M . Machine learning improves mortality risk prediction after cardiac surgery: systematic review and meta-analysis. J Thorac Cardiovasc Surg. (2020). 10.1016/j.jtcvs.2020.07.105 32900480

[10] Desai RJ Wang SV Vaduganathan M Evers T Schneeweiss S. Comparison of machine learning methods with traditional models for use of administrative claims with electronic medical records to predict heart failure outcomes. JAMA Network Open. (2020) 3:e1918962. 10.1001/jamanetworkopen.2019.18962 31922560 PMC6991258

[11] Christodoulou E Ma J Collins GS Steyerberg EW Verbakel JY Van Calster B . systematic review shows no performance benefit of machine learning over logistic regression for clinical prediction models. J Clin Epidemiol. (2019) 110:12llmio 10.1016/j.jclinepi.2019.02.004 30763612

[12] Frizzell JD Liang L Schulte PJ Yancy CW Heidenreich PA Hernandez AF . Prediction of 30-day all-cause readmissions in patients hospitalized for heart failure comparison of machine learning and other statistical approaches. JAMA Cardiology. (2017) 2:12–22. 10.1001/jamacardio.2016.3956 27784047

[13] Wynants L Van Calster B Bonten MMJ Riley RD Heinze G Schuit E . Prediction models for diagnosis and prognosis of covid-19 infection: systematic review and critical appraisal. BMJ. (2020) 369:m1328. 10.1101/2020.03.24.20041020 32265220 PMC7222643

[14] Alba AC Agoritsas T Jankowski M Courvoisier D Walter SD Guyatt GH . Risk prediction models for mortality in ambulatory patients with heart failure a systematic review. Circ-Heart Fail. (2013) 6:881–9. 10.1161/CIRCHEARTFAILURE.112.000043 23888045

[15] Michaud AM Parker SIA Ganshorn H Ezekowitz JA McRae AD. Prediction of early adverse events in emergency department patients with acute heart failure: a systematic review. Canadian J Cardiol. (2018) 34:168–79. 10.1016/j.cjca.2017.09.004 29287944

[16] Echouffo-Tcheugui JB Greene SJ Papadimitriou L Zannad F Yancy CW Gheorghiade M . Population risk prediction models for incident heart failure a systematic review. Circ-Heart Failure. (2015) 8:438–47. 10.1161/CIRCHEARTFAILURE.114.001896 25737496

[17] Bazoukis G Stavrakis S Zhou J Bollepalli SC Tse G Zhang Q . Machine learning vs. conventional clinical methods in guiding management of heart failure patients-a systematic review. Heart Fail Rev. (2020) 26:23–34. 10.1007/s10741-020-10007-3 32720083 PMC7384870

[18] Di Tanna GL Wirtz H Burrows KL Globe G. Evaluating risk prediction models for adults with heart failure: a systematic literature review. Plos ONE. (2020) 15:e0224135. 10.1371/journal.pone.0235970 31940350 PMC6961879

[19] Rahimi K Bennett D Conrad N Williams TM Basu J Dwight J . Risk prediction in patients with heart failure a systematic review and analysis. Jacc-Heart Failure. (2014) 2:440–6. 10.1016/j.jchf.2014.04.008 25194291

[20] Moher D Liberati A Tetzlaff J Altman DG PRISMA Group. Preferred reporting items for systematic reviews and meta-analyses: the PRISMA statement. Bmj. (2009) 339:b2535. 10.1136/bmj.b2535 19622551 PMC2714657

[21] Moons KG de Groot JA Bouwmeester W Vergouwe Y Mallett S Altman DG . Critical appraisal and data extraction for systematic reviews of prediction modelling studies: the CHARMS checklist. PLoS Med. (2014) 11:e1001744. 10.1371/journal.pmed.1001744 25314315 PMC4196729

[22] Debray TP Damen JA Riley RD Snell K Reitsma JB Hooft L . A framework for meta-analysis of prediction model studies with binary and time-to-event outcomes. Stat Methods Med Res. (2019) 28:2768–86. 10.1177/0962280218785504 30032705 PMC6728752

[23] Gage BF van Walraven C Pearce L Hart RG Koudstaal PJ Petersen P. Selecting patients with atrial fibrillation for anticoagulation - Stroke risk stratification in patients taking aspirin. Circulation. (2004) 110:2287–92. 10.1161/01.CIR.0000145172.55640.93 15477396

[24] MedCalc Statistical Software version 19,.2.6 MedCalc Software bv, Ostend, Belgium; Available online at: https://www.medcalc.org (2020).

[25] Altman DG Royston P. The cost of dichotomising continuous variables. Bmj. (2006) 332:1080. 10.1136/bmj.332.7549.1080 16675816 PMC1458573

[26] Harrell FE Lee KL Mark DB. Multivariable prognostic models: issues in developing models, evaluating assumptions and adequacy, and measuring and reducing errors. Stat Med. (1996) 15:361–87. 10.1002/(SICI)1097-0258(19960229)15:4<361::AID-SIM168>3.0.CO;2-4 8668867

[27] Gomes HM Barddal JP Enembreck F Bifet A. A survey on ensemble learning for data stream classification. Acm Comput Surv. (2017) 50:1–36. 10.1145/3054925

[28] Esteva A Robicquet A Ramsundar B Kuleshov V DePristo M Chou K . A guide to deep learning in healthcare. Nat Med. (2019) 25:24–9. 10.1038/s41591-018-0316-z 30617335

[29] Wolff RF Moons KG Riley RD Whiting PF Westwood M Collins GS et al. PROBAST: a tool to assess the risk of bias and applicability of prediction model studies. Ann Intern Med. (2019) 170:51–8. 10.7326/M18-1376 30596875

[30] Shickel B Tighe PJ Bihorac A Rashidi P. Deep EHR: a survey of recent advances in deep learning techniques for electronic health record (EHR) analysis. IEEE J Biomed Health Informat. (2018) 22:1589–604. 10.1109/JBHI.2017.2767063 29989977 PMC6043423

[31] Huang ZX Chan TM Dong W. MACE prediction of acute coronary syndrome via boosted resampling classification using electronic medical records. J Biomed Inform. (2017) 66:161–70. 10.1016/j.jbi.2017.01.001 28065840

