# Supplementary material for: Comparing Machine Learning Models and Statistical Models for Predicting Heart Failure Events: A Systematic Review and Meta-Analysis
Source: Front Cardiovasc Med. 2022 Apr 6;9:812276. doi: 10.3389/fcvm.2022.812276 (PMC9020815; doi:10.3389/fcvm.2022.812276)
Supplement: Supplementary file 1 [file Data_Sheet_1.ZIP › Supplementary Material.docx]

**Supplementary Material**

**Content**

[A Predictor Distribution 1](#_Toc82109832)

[B Predictor Usage 2](#_Toc82109833)

[C List of included models 3](#_Toc82109834)

[D Reference 14](#_Toc82109835)

# Predictor Distribution


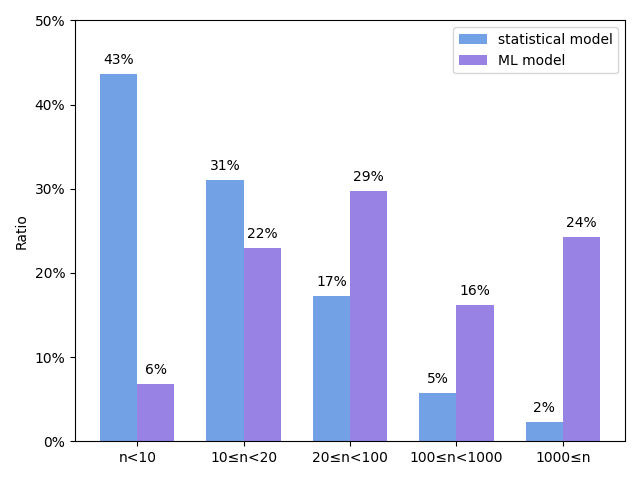


Supplement Figure 1. Predictor distribution

# Predictor Usage


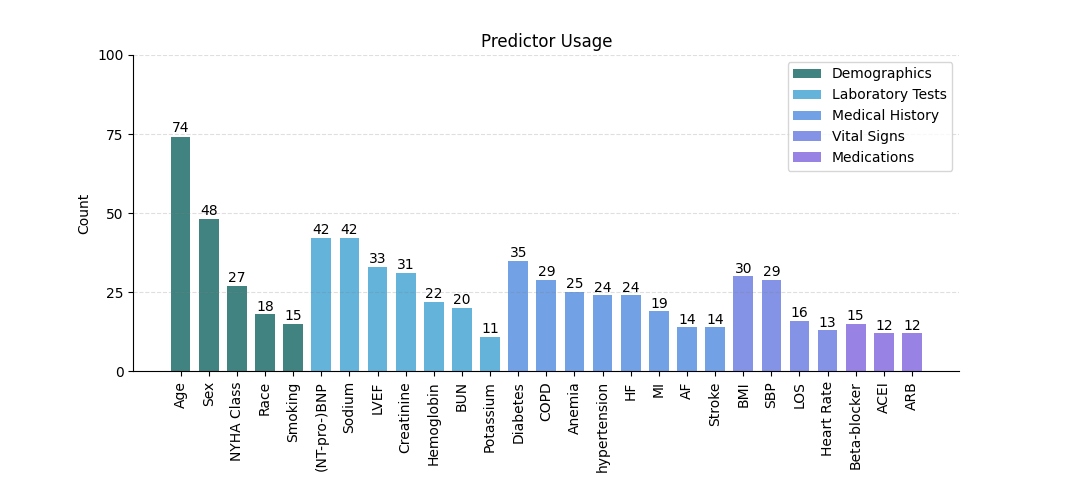


Supplementary Figure 1. Predictor usage

NYHA: New York Heart Association, BNP: B-type Natriuretic Peptide, NT-pro-BNP: N-terminal Pro BNP, LVEF: Left Ventricular Ejection Fraction, BUN: Blood Urea Nitrogen, BMI: Body Mass Index, SBP: Systolic Blood Pressure, LOS: Length of Stay, COPD: Chronic Obstructive Pulmonary Disease, MI: Myocardial Infarction, AF: Atrial Fibrillation, ARB: Angiotensin Receptor Blocker, ACEI: Angiotensin-Converting Enzyme Inhibitor

# List of included models

Supplementary Table 1. List of included models

| No | First Author | Reference  No. | Study Type | Model Name | Predicted Outcome | Follow Up Time * | Model  Category | C-Index | CI |
| --- | --- | --- | --- | --- | --- | --- | --- | --- | --- |
| 1 | Kasahara, Shintaro | 1 | Develop | 3A3B | Mortality | 5.7Y | Cox | 0.71 | 0.03 |
| 2 | Betihavas, Vasiliki | 2 | Develop |  | Readmission | 1Y | Cox | 0.80 | 0.06 |
| 3 | Scrutinio, Domenico | 3 | Validation | ADHF/NT-pro-BNP | Mortality | 1Y | Score | 0.74 | 0.04 |
| 4 | Scrutinio, Domenico | 3 | Validation | SHFM | Mortality | 1Y | Score | 0.74 | 0.04 |
| 5 | Scrutinio, Domenico | 4 | Develop |  | Mortality | 1Y | LR | 0.84 | 0.03 |
| 6 | Mortazavi, Bobak J. | 5 | Develop |  | Readmission | 6M | RF | 0.65 | 0.01 |
| 7 | Mortazavi, Bobak J. | 5 | Develop |  | Readmission | 1M | RF | 0.63 | 0.01 |
| 8 | Mortazavi, Bobak J. | 5 | Develop |  | Readmission | 1M | Boosting | 0.60 | 0.01 |
| 9 | Mortazavi, Bobak J. | 5 | Develop |  | Readmission | 6M | LR | 0.57 | 0.01 |
| 10 | Mortazavi, Bobak J. | 5 | Develop |  | Readmission | 1M | LR | 0.53 | 0.01 |
| 11 | Formiga, Francesc | 6 | Validation | RR Score | Readmission | 1M | LR | 0.65 | 0.07 |
| 12 | Formiga, Francesc | 6 | Validation | RR Score | Readmission | 3M | LR | 0.62 | 0.06 |
| 13 | Gorodeski, Eiran Z. | 7 | Validation | SHFM | Mortality | 1Y | Cox | 0.71 | 0.06 |
| 14 | Gorodeski, Eiran Z. | 7 | Validation | SHFM | Mortality | 2Y | Cox | 0.67 | 0.06 |
| 15 | Circulation Journal | 8 | Validation | SHFM | Mortality | 1Y | Cox | 0.66 | 0.01 |
| 16 | Circulation Journal | 8 | Validation | SHFM | Mortality | 1Y | Cox | 0.65 | 0.01 |
| 17 | Amarasingham, Ruben | 9 | Develop |  | Readmission | 1M | LR | 0.72 | 0.02 |
| 18 | Amarasingham, Ruben | 9 | Develop |  | Mortality | 1M | LR | 0.86 | 0.02 |
| 19 | Amarasingham, Ruben | 9 | Validation | Tabak | Mortality | 1M | Score | 0.84 | 0.02 |
| 20 | Amarasingham, Ruben | 9 | Validation | CMS | Readmission | 1M | Score | 0.66 | 0.03 |
| 21 | Amarasingham, Ruben | 9 | Validation | ADHERE | Mortality | 1M | Score | 0.73 | 0.02 |
| 22 | Amarasingham, Ruben | 9 | Validation | Tabak | Readmission | 1M | Score | 0.61 | 0.02 |
| 23 | Amarasingham, Ruben | 9 | Validation | CMS | Mortality | 1M | Score | 0.72 | 0.02 |
| 24 | Amarasingham, Ruben | 9 | Validation | ADHERE | Readmission | 1M | Score | 0.56 | 0.02 |
| 25 | Mahajan, Satish M. | 10 | Develop |  | Readmission | 1M | LR | 0.63 | 0.01 |
| 26 | Nunez, Julio | 11 | Develop |  | Mortality | 1Y | LR | 0.72 | 0.03 |
| 27 | Scrutinio, Domenico | 12 | Develop | ADHF/NT-pro-BNP | Mortality | 1Y | Score | 0.77 | 0.06 |
| 28 | Mahajan, Satish M. | 13 | Develop |  | Readmission | 1M | LR | 0.64 | 0.01 |
| 29 | Freitas, Pedro | 14 | Validation | SHFM | Mortality | 1Y | Score | 0.75 | 0.06 |
| 30 | Freitas, Pedro | 14 | Validation | HFSS | Mortality | 1Y | Cox | 0.71 | 0.06 |
| 31 | Freitas, Pedro | 14 | Validation | AMAGGIC | Mortality | 1Y | Score | 0.74 | 0.06 |
| 32 | Freitas, Pedro | 14 | Validation | MECKI | Mortality | 1Y | Cox | 0.74 | 0.06 |
| 33 | Freitas, Pedro | 14 | Validation | SHFM | Mortality | 2Y | Score | 0.76 | 0.05 |
| 34 | Freitas, Pedro | 14 | Validation | HFSS | Mortality | 2Y | Score | 0.70 | 0.06 |
| 35 | Freitas, Pedro | 14 | Validation | AMAGGIC | Mortality | 2Y | Score | 0.76 | 0.06 |
| 36 | Freitas, Pedro | 14 | Validation | MECKI | Mortality | 2Y | Score | 0.78 | 0.06 |
| 37 | Desai, Rishi J. | 15 | Develop |  | Mortality | 1Y | Boosting | 0.77 | 0.02 |
| 38 | Desai, Rishi J. | 15 | Develop |  | Mortality | 1Y | RF | 0.76 | 0.02 |
| 39 | Desai, Rishi J. | 15 | Develop |  | Mortality | 1Y | LASSO | 0.75 | 0.02 |
| 40 | Desai, Rishi J. | 15 | Develop |  | Mortality | 1Y | LR | 0.75 | 0.02 |
| 41 | Desai, Rishi J. | 15 | Develop |  | Mortality | 1Y | DT | 0.70 | 0.02 |
| 42 | Giulia Lorenzoni | 16 | Develop |  | Readmission | 1Y | LR | 0.63 | 0.01 |
| 43 | Giulia Lorenzoni | 16 | Develop |  | Readmission | 1Y | L1+L2 | 0.62 | 0.03 |
| 44 | Giulia Lorenzoni | 16 | Develop |  | Readmission | 1Y | CART | 0.58 | 0.03 |
| 45 | Giulia Lorenzoni | 16 | Develop |  | Readmission | 1Y | RF | 0.67 | 0.03 |
| 46 | Giulia Lorenzoni | 16 | Develop |  | Readmission | 1Y | L-Boosting | 0.65 | 0.03 |
| 47 | Giulia Lorenzoni | 16 | Develop |  | Readmission | 1Y | A-Boosting | 0.64 | 0.03 |
| 48 | Giulia Lorenzoni | 16 | Develop |  | Readmission | 1Y | SVM | 0.62 | 0.03 |
| 49 | Giulia Lorenzoni | 16 | Develop |  | Readmission | 1Y | BB | 0.64 | 0.03 |
| 50 | Hiroki Nakano | 17 | Validation | GWTG-HF | Mortality | 6M | Score | 0.69 | 0.08 |
| 51 | Hiroki Nakano | 17 | Validation | OPTIMIZE-HF-IN | Mortality | 6M | Score | 0.70 | 0.09 |
| 52 | Hiroki Nakano | 17 | Validation | ADHERE | Mortality | 6M | LR | 0.69 | 0.09 |
| 53 | Hiroki Nakano | 17 | Validation | EFFECT | Mortality | 6M | Score | 0.72 | 0.08 |
| 54 | Hiroki Nakano | 17 | Validation | ASCEND | Mortality | 6M | Score | 0.70 | 0.08 |
| 55 | Hiroki Nakano | 17 | Validation | OPTIME_CHF | Mortality | 6M | Score | 0.74 | 0.08 |
| 56 | Hiroki Nakano | 17 | Validation | OPTIMIZE-HF-90 | Mortality | 6M | Score | 0.69 | 0.08 |
| 57 | Hiroki Nakano | 17 | Validation | GWTG-HF | Mortality | 1Y | Score | 0.66 | 0.07 |
| 58 | Hiroki Nakano | 17 | Validation | OPTIMIZE-HF-IN | Mortality | 1Y | Score | 0.69 | 0.06 |
| 59 | Hiroki Nakano | 17 | Validation | ADHERE | Mortality | 1Y | LR | 0.66 | 0.07 |
| 60 | Hiroki Nakano | 17 | Validation | EFFECT | Mortality | 1Y | Score | 0.73 | 0.06 |
| 61 | Hiroki Nakano | 17 | Validation | ASCEND | Mortality | 1Y | Score | 0.71 | 0.06 |
| 62 | Hiroki Nakano | 17 | Validation | OPTIME-CHF | Mortality | 1Y | Score | 0.73 | 0.06 |
| 63 | Hiroki Nakano | 17 | Validation | OPTIMIZE-HF-90 | Mortality | 1Y | Score | 0.71 | 0.06 |
| 64 | Barge-Caballero, Eduardo | 18 | Validation | MAGGIC | Mortality | 3.4Y | Score | 0.76 | 0.04 |
| 65 | Barge-Caballero, Eduardo | 18 | Validation | MAGGIC | Mortality | 3.4Y | Score | 0.71 | 0.06 |
| 66 | Landicho, Junar Arciete | 19 | Develop |  | Readmission | 1Y | SVM | 0.60 | 0.10 |
| 67 | Landicho, Junar Arciete | 19 | Develop |  | Readmission | 1Y | LR | 0.58 | 0.10 |
| 68 | Landicho, Junar Arciete | 19 | Develop |  | Readmission | 1Y | RF | 0.55 | 0.10 |
| 69 | Landicho, Junar Arciete | 19 | Develop |  | Readmission | 1Y | NN | 0.50 | 0.10 |
| 70 | Honold, Joerg | 20 | Validation | SHFM | Mortality | 3Y | Cox | 0.81 | 0.08 |
| 71 | Cox, Zachary L. | 21 | Develop |  | Readmission | 1M | LR | 0.72 | 0.03 |
| 72 | Fleming, Lisa M. | 22 | Develop |  | Readmission | 1M | Generalized estimating equations | 0.66 | 0.04 |
| 73 | Hwang, In-Chang | 23 | Develop |  | Mortality | 2Y | Cox | 0.80 | 0.03 |
| 74 | Hwang, In-Chang | 23 | Develop |  | Mortality | 2Y | Cox | 0.72 | 0.04 |
| 75 | French, Benjamin | 24 | Develop |  | Mortality | 1Y | Cox | 0.83 | 0.04 |
| 76 | Voors, Adriaan A. | 25 | Develop |  | Readmission | 1.75Y | Cox | 0.68 | 0.02 |
| 77 | Voors, Adriaan A. | 25 | Validation |  | Readmission | 1.75Y | Cox | 0.63 | 0.03 |
| 78 | Voors, Adriaan A. | 25 | Develop |  | Mortality | 1.75Y | Cox | 0.73 | 0.02 |
| 79 | Voors, Adriaan A. | 25 | Validation |  | Mortality | 1.75Y | Cox | 0.73 | 0.02 |
| 80 | Lupon, Josep | 26 | Develop |  | Mortality | 3Y | Cox | 0.79 | 0.02 |
| 81 | Ingle, Lee | 27 | Develop |  | Mortality | 8.6Y | Cox | 0.75 | 0.05 |
| 82 | Garcia-Olmos, Luis | 28 | Develop |  | Readmission | 1Y | LR | 0.71 | 0.07 |
| 83 | Tokatli, Alptug | 29 | Develop |  | Mortality | 9Y | Score | 0.70 | 0.04 |
| 84 | Tan, Bo-yu | 30 | Develop |  | Readmission | 3M | LR | 0.73 | 0.05 |
| 85 | Riegel, Barbara | 31 | Develop |  | Readmission | 6M | LR | 0.82 | 0.05 |
| 86 | Ahmad, Tariq | 32 | Develop |  | Mortality | 2.6Y | Cox | 0.80 | 0.04 |
| 87 | Sepehrvand, Nariman | 33 | Validation | EHMRG | Mortality | 1M | Score | 0.71 | 0.02 |
| 88 | Quiros-Lopez, R. | 34 | Validation | CACE-HF | Mortality | 1Y | Score | 0.67 | 0.02 |
| 89 | Salah, Khibar | 35 | Validation | ELAN-HF | Mortality | 6M | Score | 0.77 | 0.08 |
| 90 | Wussler, Desiree | 36 | Validation | MESSI | Mortality | 1M | Score | 0.80 | 0.04 |
| 91 | O'Connor, Christopher M. | 37 | Develop | HF-Action | Mortality | 2.5Y | Score | 0.70 | 0.03 |
| 92 | Wang, Zhe | 38 | Develop |  | Mortality | 1Y | Boosting | 0.90 | 0.01 |
| 93 | Wang, Zhe | 38 | Develop |  | Mortality | 1Y | RF | 0.89 | 0.01 |
| 94 | Wang, Zhe | 38 | Develop |  | Mortality | 1Y | DL | 0.88 | 0.01 |
| 95 | Wang, Zhe | 38 | Develop |  | Mortality | 1Y | NN | 0.88 | 0.01 |
| 96 | Wang, Zhe | 38 | Develop |  | Mortality | 1Y | Boosting | 0.88 | 0.01 |
| 97 | Wang, Zhe | 38 | Develop |  | Mortality | 1Y | NN | 0.85 | 0.01 |
| 98 | Wang, Zhe | 38 | Develop |  | Mortality | 1Y | SVM | 0.81 | 0.01 |
| 99 | Wang, Zhe | 38 | Develop |  | Mortality | 1Y | LR | 0.78 | 0.01 |
| 100 | Wang, Zhe | 38 | Develop |  | Mortality | 1M | DL | 0.89 | 0.01 |
| 101 | Wang, Zhe | 38 | Develop |  | Mortality | 1M | NN | 0.85 | 0.01 |
| 102 | Wang, Zhe | 38 | Develop |  | Mortality | 1M | Boosting | 0.84 | 0.01 |
| 103 | Wang, Zhe | 38 | Develop |  | Mortality | 1M | RF | 0.83 | 0.01 |
| 104 | Wang, Zhe | 38 | Develop |  | Mortality | 1M | Boosting | 0.83 | 0.01 |
| 105 | Wang, Zhe | 38 | Develop |  | Mortality | 1M | NN | 0.81 | 0.01 |
| 106 | Wang, Zhe | 38 | Develop |  | Mortality | 1M | SVM | 0.78 | 0.02 |
| 107 | Wang, Zhe | 38 | Develop |  | Mortality | 1M | LR | 0.74 | 0.02 |
| 108 | Koller, Lorenz | 39 | Develop |  | Mortality | 3.7Y | Cox | 0.70 | 0.04 |
| 109 | Mendez Fernandez, Ana Belen | 40 | Develop |  | Mortality | 1.25Y | Cox | 0.82 | 0.05 |
| 110 | Roset, Alex | 41 | Develop |  | Mortality | 1M | Cox | 0.70 | 0.05 |
| 111 | Zhang, Jufen | 42 | Develop |  | Mortality | 1M | DT | 0.89 | 0.04 |
| 112 | Zhang, Jufen | 42 | Develop |  | Mortality | 1M | LR | 0.86 | 0.05 |
| 113 | Jackson, Colette E. | 43 | Develop |  | Mortality | 3.2Y | Cox | 0.73 | 0.04 |
| 114 | Hammill, Bradley G. | 44 | Develop |  | Mortality | 1M | GLM | 0.76 | 0.01 |
| 115 | Hammill, Bradley G. | 44 | Develop |  | Readmission | 1M | GLM | 0.60 | 0.01 |
| 116 | Bulluck, Heerajnarain | 45 | Develop |  | Readmission | 1Y | LR | 0.87 | 0.02 |
| 117 | Bulluck, Heerajnarain | 45 | Validation |  | Readmission | 1Y | LR | 0.86 | 0.03 |
| 118 | Chen, Peipei | 46 | Develop |  | Readmission | 1Y | DL | 0.69 | 0.09 |
| 119 | Chen, Peipei | 46 | Develop |  | Readmission | 1Y | LR | 0.68 | 0.08 |
| 120 | Chen, Peipei | 46 | Develop |  | Readmission | 1Y | NN | 0.68 | 0.08 |
| 121 | Chen, Peipei | 46 | Develop |  | Readmission | 1Y | DL | 0.66 | 0.07 |
| 122 | Reeder, Harrison T. | 47 | Develop |  | Mortality | 3.5Y | Cox | 0.79 | 0.04 |
| 123 | Jing, Linyuan | 48 | Develop |  | Mortality | 1Y | Boosting | 0.77 | 0.01 |
| 124 | Jing, Linyuan | 48 | Develop |  | Mortality | 1Y | RF | 0.76 | 0.01 |
| 125 | Jing, Linyuan | 48 | Develop |  | Mortality | 1Y | LR | 0.74 | 0.01 |
| 126 | Chicco, Davide | 49 | Develop |  | Mortality | 4M | RF | 0.80 | 0.05 |
| 127 | Chicco, Davide | 49 | Develop |  | Mortality | 4M | Boosting | 0.75 | 0.05 |
| 128 | Chicco, Davide | 49 | Develop |  | Mortality | 4M | SVM | 0.75 | 0.05 |
| 129 | Chicco, Davide | 49 | Develop |  | Mortality | 4M | DT | 0.68 | 0.06 |
| 130 | Chicco, Davide | 49 | Develop |  | Mortality | 4M | LR | 0.64 | 0.06 |
| 131 | Chicco, Davide | 49 | Develop |  | Mortality | 4M | Others | 0.64 | 0.06 |
| 132 | Chicco, Davide | 49 | Develop |  | Mortality | 4M | NB | 0.59 | 0.07 |
| 133 | Chicco, Davide | 49 | Develop |  | Mortality | 4M | NN | 0.56 | 0.07 |
| 134 | Chicco, Davide | 49 | Develop |  | Mortality | 4M | KNN | 0.49 | 0.07 |
| 135 | Golas, Sara Bersche | 50 | Develop |  | Readmission | 1M | DL | 0.71 | 0.03 |
| 136 | Golas, Sara Bersche | 50 | Develop |  | Readmission | 1M | DL | 0.70 | 0.03 |
| 137 | Golas, Sara Bersche | 50 | Develop |  | Readmission | 1M | L2 | 0.66 | 0.03 |
| 138 | Golas, Sara Bersche | 50 | Develop |  | Readmission | 1M | Boosting | 0.65 | 0.02 |
| 139 | Angraal, Suveen | 51 | Develop |  | Mortality | 3Y | RF | 0.72 | 0.03 |
| 140 | Angraal, Suveen | 51 | Develop |  | Mortality | 3Y | Boosting | 0.68 | 0.02 |
| 141 | Angraal, Suveen | 51 | Develop |  | Mortality | 3Y | LR | 0.66 | 0.03 |
| 142 | Angraal, Suveen | 51 | Develop |  | Mortality | 3Y | SVM | 0.66 | 0.06 |
| 143 | Angraal, Suveen | 51 | Develop |  | Mortality | 3Y | LASSO | 0.65 | 0.04 |
| 144 | Angraal, Suveen | 51 | Develop |  | Mortality | 1Y | RF | 0.69 | 0.06 |
| 145 | Angraal, Suveen | 51 | Develop |  | Mortality | 1Y | Boosting | 0.65 | 0.06 |
| 146 | Angraal, Suveen | 51 | Develop |  | Mortality | 1Y | LR | 0.68 | 0.14 |
| 147 | Angraal, Suveen | 51 | Develop |  | Mortality | 1Y | SVM | 0.69 | 0.08 |
| 148 | Angraal, Suveen | 51 | Develop |  | Mortality | 1Y | LASSO | 0.70 | 0.08 |
| 149 | Canepa, Marco | 52 | Validation | MAGGIC | Mortality | 3Y | Score | 0.73 | 0.01 |
| 150 | Wang, Zhe | 53 | Develop |  | Mortality | 1Y | Others | 0.85 | 0.02 |
| 151 | Wang, Zhe | 53 | Develop |  | Mortality | 1M | Others | 0.89 | 0.01 |
| 152 | Vakil, Kairav P. | 54 | Develop | Modified SHFM | Mortality | 6M | Cox | 0.84 | 0.06 |
| 153 | Fontanive, Paolo | 55 | Develop |  | Mortality | 2.5Y | Cox | 0.78 | 0.03 |
| 154 | Fontanive, Paolo | 55 | Validation | MUSIC | Mortality | 2.5Y | Score | 0.72 | 0.03 |
| 155 | Fontanive, Paolo | 55 | Validation | 3CHF | Mortality | 2.5Y | Score | 0.72 | 0.03 |
| 156 | Bowen, Garrett S. | 56 | Develop |  | Mortality | 1Y | Cox | 0.68 | 0.01 |
| 157 | Bowen, Garrett S. | 56 | Develop |  | Mortality | 2Y | Cox | 0.67 | 0.01 |
| 158 | Bowen, Garrett S. | 56 | Develop |  | Mortality | 1M | Cox | 0.70 | 0.02 |
| 159 | Han, Qiang | 57 | Develop |  | Readmission | 1Y | Cox | 0.74 | 0.06 |
| 160 | Escobar, Antonio | 58 | Develop |  | Mortality | 1Y | LR | 0.70 | 0.03 |
| 161 | Hung, Wei-Kai | 59 | Develop |  | Mortality | 1Y | Score | 0.68 | 0.04 |
| 162 | Hung, Wei-Kai | 59 | Validation | 3CHF | Mortality | 1Y | Score | 0.64 | 0.04 |
| 163 | Canepa, Marco | 60 | Validation | MAGGIC | Mortality | 1Y | Score | 0.74 | 0.02 |
| 164 | Canepa, Marco | 60 | Validation | GISSI-HF | Mortality | 1Y | Score | 0.74 | 0.02 |
| 165 | Canepa, Marco | 60 | Validation | CHARM | Mortality | 1Y | Score | 0.73 | 0.02 |
| 166 | Canepa, Marco | 60 | Validation | SHFM | Mortality | 1Y | Cox | 0.71 | 0.02 |
| 167 | Vishram-Nielsen, Julie K. K. | 61 | Validation | SHFM | Mortality | 1Y | Cox | 0.76 | 0.05 |
| 168 | Vishram-Nielsen, Julie K. K. | 61 | Validation | SHFM | Mortality | 3Y | Score | 0.73 | 0.04 |
| 169 | Vishram-Nielsen, Julie K. K. | 61 | Validation | MAGGIC | Mortality | 1Y | Score | 0.71 | 0.05 |
| 170 | Vishram-Nielsen, Julie K. K. | 61 | Validation | MAGGIC | Mortality | 3Y | Score | 0.69 | 0.04 |
| 171 | Sawano, Mitsuaki | 62 | Validation | MAGGIC | Mortality | 1Y | Score | 0.71 | 0.04 |
| 172 | Sawano, Mitsuaki | 62 | Update | Modified MAGGIC | Mortality | 1Y | Score | 0.69 | 0.04 |
| 173 | Smith, Tim | 63 | Validation | SHFM | Mortality | 1Y | Cox | 0.79 | 0.07 |
| 174 | Smith, Tim | 63 | Validation | SHFM | Mortality | 5Y | Cox | 0.71 | 0.07 |
| 175 | Chamberlain, Alanna M. | 64 | Develop |  | Mortality | 2.3Y | Cox | 0.76 | 0.05 |
| 176 | Li, Lin | 65 | Develop |  | Readmission | 1M | LR | 0.63 | 0.01 |
| 177 | Li, Lin | 65 | Develop |  | Mortality | 1M | LR | 0.71 | 0.01 |
| 178 | Miro, Oscar | 66 | Develop |  | Mortality | 1M | LR | 0.83 | 0.03 |
| 179 | Senni, Michele | 67 | Develop | 3CHF | Mortality | 1Y | Score | 0.82 | 0.01 |
| 180 | Thorvaldsen, Tonje | 68 | Develop |  | Readmission | 1M | LR | 0.73 | 0.03 |
| 181 | Thorvaldsen, Tonje | 68 | Develop |  | Readmission | 1Y | LR | 0.71 | 0.02 |
| 182 | Mahajan, Satish M. | 69 | Develop |  | Readmission | 1M | Boosting | 0.72 | 0.02 |
| 183 | Mahajan, Satish M. | 69 | Develop |  | Readmission | 1M | Boosting | 0.61 | 0.02 |
| 184 | Wang, Li | 70 | Develop |  | Readmission | 1M | LR | 0.82 | 0.01 |
| 185 | Wang, Li | 70 | Develop |  | Readmission | 1Y | LR | 0.82 | 0.01 |
| 186 | Wang, Li | 70 | Develop |  | Mortality | 1Y | LR | 0.76 | 0.01 |
| 187 | Wang, Li | 70 | Develop |  | Mortality | 1M | LR | 0.80 | 0.01 |
| 188 | Scrutinio, Domenico | 71 | Update | ADHF/NT-proBNP | Mortality | 3M | Score | 0.81 | 0.04 |
| 189 | Scrutinio, Domenico | 71 | Update | EHFS | Mortality | 3M | Score | 0.76 | 0.05 |
| 190 | Sartipy, Ulrik | 72 | Validation | MAGGIC | Mortality | 3Y | Score | 0.74 | 0.01 |
| 191 | Au, Anita G. | 73 | Validation | Keenan | Mortality | 1M | LR | 0.71 | 0.01 |
| 192 | Au, Anita G. | 73 | Validation | Keenan | Readmission | 1M | LR | 0.58 | 0.01 |
| 193 | Au, Anita G. | 73 | Validation | LaCE | Readmission | 1M | Score | 0.58 | 0.01 |
| 194 | Au, Anita G. | 73 | Validation | Krumholz | Readmission | 1M | Score | 0.55 | 0.01 |
| 195 | Au, Anita G. | 73 | Validation | Krumholz | Mortality | 1M | Score | 0.62 | 0.01 |
| 196 | Au, Anita G. | 73 | Validation | LaCE | Mortality | 1M | Score | 0.61 | 0.01 |
| 197 | Frizzell, Jarrod D. | 74 | Develop |  | Readmission | 1M | LR | 0.62 | 0.01 |
| 198 | Frizzell, Jarrod D. | 74 | Develop |  | Readmission | 1M | LR | 0.59 | 0.01 |
| 199 | Frizzell, Jarrod D. | 74 | Develop |  | Readmission | 1M | LASSO | 0.62 | 0.01 |
| 200 | Frizzell, Jarrod D. | 74 | Develop |  | Readmission | 1M | Bayesian | 0.62 | 0.01 |
| 201 | Wang, Nelson | 75 | Develop |  | Mortality | 1Y | LR | 0.65 | 0.02 |
| 202 | Cleland, John G. | 76 | Develop |  | Mortality | 1M | LR | 0.77 | 0.04 |
| 203 | Cleland, John G. | 76 | Develop |  | Mortality | 6M | LR | 0.72 | 0.03 |
| 204 | Simpson, Joanne | 77 | Develop |  | Mortality | 1Y | Cox | 0.71 | 0.02 |
| 205 | Simpson, Joanne | 77 | Develop |  | Mortality | 2Y | Cox | 0.70 | 0.03 |
| 206 | Morbach, Caroline | 78 | Develop |  | Mortality | 6M | Cox | 0.78 | 0.06 |
| 207 | Morbach, Caroline | 78 | Develop |  | Readmission | 6M | Cox | 0.66 | 0.05 |
| 208 | Cubbon, R. M. | 79 | Validation | No.694 | Readmission | 1Y | LR | 0.81 | 0.07 |
| 209 | Cubbon, R. M. | 79 | Develop | No.694 | Readmission | 1Y | LR | 0.79 | 0.07 |
| 210 | Lee, Douglas S. | 80 | Validation | EHMRG | Mortality | 1M | Score | 0.81 | 0.06 |
| 211 | Ashfaq, Awais | 81 | Develop |  | Readmission | 1M | DL | 0.77 | 0.01 |
| 212 | Xiao, Cao | 82 | Develop |  | Readmission | 1M | DL | 0.61 | 0.01 |
| 213 | Xiao, Cao | 81 | Develop |  | Readmission | 1M | LR | 0.59 | 0.01 |
| 214 | Jiang, Wei | 83 | Develop |  | Readmission | 1M | LR | 0.73 | 0.16 |
| 215 | Wessler, Benjamin S. | 84 | Validation | OPTIME | Mortality | 2M | Score | 0.72 | 0.03 |
| 216 | Wessler, Benjamin S. | 84 | Validation | EFFECT | Mortality | 1Y | Score | 0.66 | 0.02 |
| 217 | Stampehl, Mark | 85 | Develop |  | Mortality | 1M | DT | 0.73 | 0.02 |
| 218 | Stampehl, Mark | 85 | Develop |  | Mortality | 1M | DT | 0.73 | 0.02 |
| 219 | Stampehl, Mark | 85 | Develop |  | Mortality | 1M | LR | 0.75 | 0.02 |
| 220 | Stampehl, Mark | 85 | Develop |  | Mortality | 1M | LR | 0.76 | 0.02 |
| 221 | Stampehl, Mark | 85 | Develop |  | Mortality | 1Y | DT | 0.71 | 0.02 |
| 222 | Stampehl, Mark | 85 | Develop |  | Mortality | 1Y | DT | 0.71 | 0.02 |
| 223 | Stampehl, Mark | 85 | Develop |  | Mortality | 1Y | LR | 0.74 | 0.02 |
| 224 | Stampehl, Mark | 85 | Develop |  | Mortality | 1Y | LR | 0.75 | 0.02 |
| 225 | Andersson, Charlotte | 86 | Develop |  | Mortality | 1M | Score | 0.79 | 0.01 |
| 226 | Leong, Kui Toh Gerard | 87 | Develop |  | Readmission | 1M | LR | 0.76 | 0.03 |
| 227 | Novack, Victor | 88 | Develop |  | Mortality | 1Y | LR | 0.76 | 0.04 |
| 228 | Novack, Victor | 88 | Develop |  | Mortality | 1M | LR | 0.82 | 0.03 |
| 229 | Clemens, Marcell | 89 | Validation | SHFM | Mortality | 2Y | Cox | 0.79 | 0.06 |
| 230 | Clemens, Marcell | 89 | Validation | SHFM | Mortality | 5Y | Cox | 0.76 | 0.01 |
| 231 | Clemens, Marcell | 89 | Validation | SHFM | Mortality | 1Y | Cox | 0.74 | 0.08 |
| 232 | Karauzum, Kurtulus | 90 | Develop |  | Mortality | 1Y | LR | 0.74 | 0.04 |
| 233 | Yap, Jonathan | 91 | Develop |  | Mortality | 1Y | Cox | 0.67 | 0.05 |
| 234 | Yap, Jonathan | 91 | Develop |  | Mortality | 1Y | Cox | 0.68 | 0.04 |
| 235 | Yap, Jonathan | 91 | Develop |  | Mortality | 2Y | Cox | 0.63 | 0.05 |
| 236 | Yap, Jonathan | 91 | Develop |  | Mortality | 2Y | Cox | 0.65 | 0.04 |
| 237 | Felker, G. Michael | 92 | Develop |  | Readmission | 3.7Y | Cox | 0.76 | 0.02 |
| 238 | Felker, G. Michael | 92 | Develop |  | Mortality | 3.7Y | Cox | 0.77 | 0.01 |
| 239 | O'Connor, Christopher M. | 93 | Develop | ESCAPE | Mortality | 6M | Score | 0.78 | 0.05 |
| 240 | Nakazone, Marcelo Arruda | 94 | Validation | CALL | Mortality | 4Y | Cox | 0.69 | 0.01 |
| 241 | Miro, Oscar | 95 | Validation | MESSI | Mortality | 1M | Score | 0.81 | 0.02 |
| 242 | Mahajan, Satish M. | 96 | Develop |  | Readmission | 1M | DT | 0.70 | 0.01 |
| 243 | Mahajan, Satish M. | 96 | Develop |  | Readmission | 1M | LR | 0.69 | 0.01 |
| 244 | Montero-Perez-Barquero, Manuel | 97 | Validation | SENIORS | Mortality | 1Y | Score | 0.66 | 0.04 |
| 245 | Mahajan, Satish M. | 98 | Develop |  | Readmission | 1M | LASSO | 0.77 | 0.01 |
| 246 | Shiraishi, Yasuyuki | 99 | Validation | SHFM | Mortality | 1Y | Cox | 0.75 | 0.02 |
| 247 | Shiraishi, Yasuyuki | 99 | Validation | SHFM | Mortality | 1Y | Cox | 0.69 | 0.02 |
| 248 | Quan, Huynh | 100 | Validation |  | Readmission | 1M | Score | 0.73 | 0.03 |
| 249 | Quan, Huynh | 100 | Validation |  | Mortality | 1M | Score | 0.85 | 0.03 |
| 250 | Regoli, Francois | 101 | Validation | SHFM | Mortality | 3.3Y | Cox | 0.64 | 0.03 |
| 251 | Khanam, Sayma Sabrina | 102 | Validation | MAGGIC | Mortality | 1Y | Score | 0.73 | 0.01 |
| 252 | Sudhakar, Selvin | 103 | Validation | RR Score | Readmission | 1M | Score | 0.62 | 0.10 |
| 253 | Nagai, Toshiyuki | 104 | Validation | ASCEND-HF | Mortality | 1M | Score | 0.66 | 0.07 |
| 254 | Nagai, Toshiyuki | 104 | Validation | OPTIME-CHF | Mortality | 3M | Score | 0.70 | 0.05 |
| 255 | Nagai, Toshiyuki | 104 | Validation | ASCEND-HF | Mortality | 6M | Score | 0.69 | 0.04 |
| 256 | Nagai, Toshiyuki | 104 | Validation | ASCEND-HF | Mortality | 1M | Score | 0.75 | 0.07 |
| 257 | Nagai, Toshiyuki | 104 | Validation | OPTIME-CHF | Mortality | 3M | Score | 0.75 | 0.05 |
| 258 | Nagai, Toshiyuki | 104 | Validation | ASCEND-HF | Mortality | 6M | Score | 0.75 | 0.04 |
| 259 | Li, Song | 105 | Validation | SHFM | Mortality | 1Y | Cox | 0.67 | 0.04 |
| 260 | Li, Song | 105 | Validation | SHFM | Mortality | 1Y | Cox | 0.79 | 0.04 |
| 261 | Laszczynska, Olga | 106 | Validation | SHFM | Mortality | 1Y | Cox | 0.79 | 0.09 |
| 262 | Laszczynska, Olga | 106 | Validation | SHFM | Mortality | 5Y | Cox | 0.74 | 0.07 |
| 263 | Laszczynska, Olga | 106 | Validation | SHFM | Mortality | 3Y | Cox | 0.73 | 0.07 |
| 264 | Falletta, Calogero | 107 | Develop |  | Mortality | 2.2Y | Cox | 0.68 | 0.07 |
| 265 | Nunez, Julio | 108 | Develop |  | Mortality | 1.5Y | Cox | 0.79 | 0.05 |
| 266 | Yuntao Chen | 109 | Develop |  | Mortality | 1Y | Cox | 0.74 | 0.03 |
| 267 | Yuntao Chen | 109 | Develop |  | Mortality | 1Y | Cox | 0.75 | 0.05 |
| 268 | Yuntao Chen | 109 | Develop |  | Mortality | 1Y | Cox | 0.78 | 0.03 |
| 269 | Hao Li | 110 | Develop |  | Mortality | 3Y | LR | 0.80 | 0.01 |
| 270 | Dimitrie Siriopol | 111 | Develop |  | Mortality | 1.5Y | Cox | 0.74 | 0.07 |
| 271 | Dimitrie Siriopol | 111 | Develop |  | Mortality | 1.5Y | DT | 0.79 | 0.07 |
| 272 | Tauben Averbuch | 111 | Develop |  | Readmission | 1M | log-binomial | 0.64 | 0.04 |
| 273 | Chengsheng Ju | 112 | Develop |  | Mortality | 1M | boosting | 0.90 | 0.04 |
| 274 | Chengsheng Ju | 112 | Develop |  | Mortality | 3M | boosting | 0.90 | 0.04 |
| 275 | Adi Elias | 113 | Develop | SOFA | Mortality | 1M | Score | 0.71 | 0.03 |
| 276 | Adi Elias | 113 | Develop | GWTG-HF | Mortality | 1M | Score | 0.75 | 0.02 |
| 277 | Nidhi Garg | 114 | Develop | EHMRG | Mortality | 7D | LR | 0.75 | 0.07 |
| 278 | Yuxi Sun | 115 | Validation | H2FPEF | Readmission | 2.3Y | Score | 0.59 | 0.05 |
| 279 | Yuxi Sun | 115 | Validation | H2FPEF | Mortality | 2.3Y | Score | 0.67 | 0.06 |
| 280 | Hao-Chih Chang | 116 | Validation |  | Mortality | 3Y | Cox | 0.66 | 0.04 |

* For the studies with censoring data, the time window indicated the average follow up time.

# Reference

| 1 | Kasahara, S., et al., The 3A3B score: The simple risk score for heart failure with preserved ejection fraction - A report from the CHART-2 Study. International Journal of Cardiology, 2019. 284: p. 42-49. |
| --- | --- |
| 2 | Betihavas, V., et al., An Absolute Risk Prediction Model to Determine Unplanned Cardiovascular Readmissions for Adults with Chronic Heart Failure. Heart Lung and Circulation, 2015. 24(11): p. 1068-1073. |
| 3 | Scrutinio, D., et al., The ADHF/NT-proBNP risk score to predict 1-year mortality in hospitalized patients with advanced decompensated heart failure. Journal of Heart and Lung Transplantation, 2014. 33(4): p. 404-411. |
| 4 | Scrutinio, D., et al., Amino-terminal pro-B-type natriuretic peptide for risk prediction in acute decompensated heart failure. Congestive heart failure (Greenwich, Conn.), 2012. 18(6): p. 308-14. |
| 5 | Mortazavi, B.J., et al., Analysis of Machine Learning Techniques for Heart Failure Readmissions. Circulation-Cardiovascular Quality and Outcomes, 2016. 9(6): p. 629-+. |
| 6 | Formiga, F., et al., Applicability of the heart failure Readmission Risk score: A first European study. International Journal of Cardiology, 2017. 236: p. 304-309. |
| 7 | Gorodeski, E.Z., et al., Application of the Seattle Heart Failure Model in Ambulatory Patients Presented to an Advanced Heart Failure Therapeutics Committee. Circulation-Heart Failure, 2010. 3(6): p. 706-+. |
| 8 | Williams, B.A. and S. Agarwal, Applying the Seattle Heart Failure Model in the Office Setting in the Era of Electronic Medical Records. Circulation Journal, 2018. 82(3): p. 724-+. |
| 9 | Amarasingham, R., et al., An Automated Model to Identify Heart Failure Patients at Risk for 30-Day Readmission or Death Using Electronic Medical Record Data. Medical Care, 2010. 48(11): p. 981-988. |
| 10 | Mahajan, S.M., et al., Can We Do More With Less While Building Predictive Models? A Study in Parsimony of Risk Models for Predicting Heart Failure Readmissions. Cin-Computers Informatics Nursing, 2019. 37(6): p. 306-314. |
| 11 | Nunez, J., et al., Clinical Role of CA125 in Worsening Heart Failure A BIOSTAT-CHF Study Subanalysis. Jacc-Heart Failure, 2020. 8(5): p. 386-397. |
| 12 | Scrutinio, D., et al., Clinical utility of N-terminal pro-B-type natriuretic peptide for risk stratification of patients with acute decompensated heart failure. Derivation and validation of the ADHF/NT-proBNP risk score. International Journal of Cardiology, 2013. 168(3): p. 2120-2126. |
| 13 | Mahajan, S.M. and R. Ghani, Combining Structured and Unstructured Data for Predicting Risk of Readmission for Heart Failure Patients. Studies in health technology and informatics, 2019. 264: p. 238-242. |
| 14 | Freitas, P., et al., Comparative Analysis of Four Scores to Stratify Patients With Heart Failure and Reduced Ejection Fraction. American Journal of Cardiology, 2017. 120(3): p. 443-449. |
| 15 | Desai, R.J., et al., Comparison of machine learning methods with traditional models for use of administrative claims with electronic medical records to predict heart failure outcomes. JAMA Network Open, 2020. 3(1): p. e1918962-e1918962. |
| 16 | Lorenzoni, G., et al., Comparison of Machine Learning Techniques for Prediction of Hospitalization in Heart Failure Patients. Journal of Clinical Medicine, 2019. 8(9). |
| 17 | Nakano, H., et al., Comparison of Mortality Prediction Models on Long-Term Mortality in Hospitalized Patients With Acute Heart Failure - The Importance of Accounting for Nutritional Status. Circulation Journal, 2019. 83(3): p. 614-+. |
| 18 | Barge-Caballero, E., et al., Comparison of predicted and observed mortality in patients with heart failure treated at a specialized unit. Revista espanola de cardiologia (English ed.), 2020. 73(8): p. 652-659. |
| 19 | Landicho, J.A., V. Esichaikul, and R.M. Sasil, Comparison of predictive models for hospital readmission of heart failure patients with cost-sensitive approach. International Journal of Healthcare Management, 2020. |
| 20 | Honold, J., et al., Comparison of the Seattle Heart Failure Model and Cardiopulmonary Exercise Capacity for Prediction of Death in Patients With Chronic Ischemic Heart Failure and Intracoronary Progenitor Cell Application. Clinical Cardiology, 2013. 36(3): p. 153-159. |
| 21 | Cox, Z.L., et al., Customizing national models for a medical center's population to rapidly identify patients at high risk of 30-day all-cause hospital readmission following a heart failure hospitalization. Heart & Lung, 2018. 47(4): p. 290-296. |
| 22 | Fleming, L.M., et al., Derivation and Validation of a 30-Day Heart Failure Readmission Model. American Journal of Cardiology, 2014. 114(9): p. 1379-1382. |
| 23 | Hwang, I.-C., et al., Derivation and validation of a mortality risk prediction model using global longitudinal strain in patients with acute heart failure. European heart journal cardiovascular Imaging, 2019. |
| 24 | French, B., et al., Development and evaluation of multi-marker risk scores for clinical prognosis. Statistical Methods in Medical Research, 2016. 25(1): p. 255-271. |
| 25 | Voors, A.A., et al., Development and validation of multivariable models to predict mortality and hospitalization in patients with heart failure. European Journal of Heart Failure, 2017. 19(5): p. 627-634. |
| 26 | Lupon, J., et al., Development of a Novel Heart Failure Risk Tool: The Barcelona Bio-Heart Failure Risk Calculator (BCN Bio-HF Calculator). Plos One, 2014. 9(1). |
| 27 | Ingle, L., et al., Development of a composite model derived from cardiopulmonary exercise tests to predict mortality risk in patients with mild-to-moderate heart failure. Heart, 2014. 100(10): p. 781-786. |
| 28 | Garcia-Olmos, L., et al., Development of a predictive model of hospitalization in primary care patients with heart failure. Plos One, 2019. 14(8). |
| 29 | Tokatli, A., et al., Discharge risk scoring method for predicting mortality in hospitalized chronic heart failure patients with severe systolic dysfunction. Acta Cardiologica, 2015. 70(4): p. 442-449. |
| 30 | Tan, B.-y., et al., Electronic medical record-based model to predict the risk of 90-day readmission for patients with heart failure. Bmc Medical Informatics and Decision Making, 2019. 19(1). |
| 31 | Riegel, B. and G.J. Knafl, Electronically monitored medication adherence predicts hospitalization in heart failure patients. Patient Preference and Adherence, 2014. 8: p. 1-13. |
| 32 | Ahmad, T., et al., Evaluation of the Incremental Prognostic Utility of Increasingly Complex Testing in Chronic Heart Failure. Circulation-Heart Failure, 2015. 8(4): p. 709-U61. |
| 33 | Sepehrvand, N., et al., External Validation and Refinement of Emergency Heart Failure Mortality Risk Grade Risk Model in Patients With Heart Failure in the Emergency Department. CJC open, 2019. 1(3): p. 123-130. |
| 34 | Quiros-Lopez, R., et al., External validation of the CACE-HF risk score for mortality in patients with heart failure. European Journal of Internal Medicine, 2019. 66: p. 35-40. |
| 35 | Salah, K., et al., External Validation of the ELAN-HF Score, Predicting 6-Month All-Cause Mortality in Patients Hospitalized for Acute Decompensated Heart Failure. Journal of the American Heart Association, 2019. 8(14). |
| 36 | Wussler, D., et al., External Validation of the MEESSI Acute Heart Failure Risk Score A Cohort Study. Annals of Internal Medicine, 2019. 170(4): p. 248-+. |
| 37 | O'Connor, C.M., et al., Factors Related to Morbidity and Mortality in Patients With Chronic Heart Failure With Systolic Dysfunction The HF-ACTION Predictive Risk Score Model. Circulation-Heart Failure, 2012. 5(1): p. 63-71. |
| 38 | Wang, Z., et al., Feature rearrangement based deep learning system for predicting heart failure mortality. Computer Methods and Programs in Biomedicine, 2020. 191. |
| 39 | Koller, L., et al., Fibroblast Growth Factor 23 Is an Independent and Specific Predictor of Mortality in Patients With Heart Failure and Reduced Ejection Fraction. Circulation-Heart Failure, 2015. 8(6): p. 1059-1067. |
| 40 | Mendez Fernandez, A.B., et al., Growth differentiation factor 15 as mortality predictor in heart failure patients with non-reduced ejection fraction. Esc Heart Failure, 2020. |
| 41 | Roset, A., et al., High-sensitivity cardiac troponin T 30 days all-come mortality in patients with acute heart failure. A Propensity Score-Matching Analysis Based on the EAHFE Registry. TROPICA4 Study. European Journal of Clinical Investigation, 2020. 50(6). |
| 42 | Zhang, J., et al., Identifying patients at risk of death or hospitalisation due to worsening heart failure using decision tree analysis: Evidence from the Trans-European Network-Home-Care Management System (TEN-HMS) Study. International Journal of Cardiology, 2013. 163(2): p. 149-156. |
| 43 | Jackson, C.E., et al., The incremental prognostic and clinical value of multiple novel biomarkers in heart failure. European Journal of Heart Failure, 2016. 18(12): p. 1491-1498. |
| 44 | Hammill, B.G., et al., Incremental Value of Clinical Data Beyond Claims Data in Predicting 30-Day Outcomes After Heart Failure Hospitalization. Circulation-Cardiovascular Quality and Outcomes, 2011. 4(1): p. 60-67. |
| 45 | Bulluck, H., et al., Independent Predictors of Cardiac Mortality and Hospitalization for Heart Failure in a Multi-Ethnic Asian ST-segment Elevation Myocardial Infarction Population Treated by Primary Percutaneous Coronary Intervention. Scientific Reports, 2019. 9. |
| 46 | Chen, P., et al., Interpretable clinical prediction via attention-based neural network. Bmc Medical Informatics and Decision Making, 2020. 20. |
| 47 | Reeder, H.T., et al., Joint Shock/Death Risk Prediction Model for Patients Considering Implantable Cardioverter-Defibrillators A Secondary Analysis of the SCD-HeFT trial. Circulation-Cardiovascular Quality and Outcomes, 2019. 12(8). |
| 48 | Jing, L., et al., A Machine Learning Approach to Management of Heart Failure Populations. Jacc-Heart Failure, 2020. 8(7): p. 578-587. |
| 49 | Chicco, D. and G. Jurman, Machine learning can predict survival of patients with heart failure from serum creatinine and ejection fraction alone. Bmc Medical Informatics and Decision Making, 2020. 20(1). |
| 50 | Golas, S.B., et al., A machine learning model to predict the risk of 30-day readmissions in patients with heart failure: a retrospective analysis of electronic medical records data. Bmc Medical Informatics and Decision Making, 2018. 18. |
| 51 | Angraal, S., et al., Machine Learning Prediction of Mortality and Hospitalization in Heart Failure With Preserved Ejection Fraction. Jacc-Heart Failure, 2020. 8(1): p. 12-21. |
| 52 | Canepa, M., et al., Modes of death and prognostic outliers in chronic heart failure. American Heart Journal, 2019. 208: p. 100-109. |
| 53 | Wang, Z., et al., Mortality prediction system for heart failure with orthogonal relief and dynamic radius means. International Journal of Medical Informatics, 2018. 115: p. 10-17. |
| 54 | Vakil, K.P., et al., Mortality prediction using a modified Seattle Heart Failure Model may improve patient selection for ventricular tachycardia ablation. American Heart Journal, 2015. 170(6): p. 1099-1104. |
| 55 | Fontanive, P., et al., A Multiparametric Clinical and Echocardiographic Score to Risk Stratify Patients with Chronic Systolic Heart Failure: Derivation and Testing. Echocardiography-a Journal of Cardiovascular Ultrasound and Allied Techniques, 2013. 30(10): p. 1172-1179. |
| 56 | Bowen, G.S., et al., A Multivariable Prediction Model for Mortality in Individuals Admitted for Heart Failure. Journal of the American Geriatrics Society, 2018. 66(5): p. 902-908. |
| 57 | Han, Q., et al., A nomogram based on a patient-reported outcomes measure: predicting the risk of readmission for patients with chronic heart failure. Health and quality of life outcomes, 2020. 18(1): p. 290-290. |
| 58 | Escobar, A., et al., A one-year mortality clinical prediction rule for patients with heart failure. European Journal of Internal Medicine, 2017. 44: p. 49-54. |
| 59 | Hung, W.-K., et al., One-Year Mortality Risk Stratification in Patients Hospitalized for Acute Decompensated Heart Failure: Construction of TSOC-HFrEF Risk Scoring Model. Acta Cardiologica Sinica, 2020. 36(3): p. 240-250. |
| 60 | Canepa, M., et al., Performance of Prognostic Risk Scores in Chronic Heart Failure Patients Enrolled in the European Society of Cardiology Heart Failure Long-Term Registry. Jacc-Heart Failure, 2018. 6(6): p. 452-462. |
| 61 | Vishram-Nielsen, J.K.K., et al., Performance of Prognostic Risk Scores in Heart Failure Patients: Do Sex Differences Exist? Canadian Journal of Cardiology, 2020. 36(1): p. 45-53. |
| 62 | Sawano, M., et al., Performance of the MAGGIC heart failure risk score and its modification with the addition of discharge natriuretic peptides. Esc Heart Failure, 2018. 5(4): p. 610-619. |
| 63 | Smith, T., et al., Performance of the Seattle Heart Failure Model in Implantable Defibrillator Patients Treated With Cardiac Resynchronization Therapy. American Journal of Cardiology, 2012. 110(3): p. 398-402. |
| 64 | Chamberlain, A.M., et al., Physical Health Status Measures Predict All-Cause Mortality in Patients With Heart Failure. Circulation-Heart Failure, 2013. 6(4): p. 669-675. |
| 65 | Li, L., et al., Predicting 30-day mortality and 30-day re-hospitalization risks in Medicare patients with heart failure discharged to skilled nursing facilities: development and validation of models using administrative data. The journal of nursing home research sciences, 2019. 5: p. 60-67. |
| 66 | Miro, O., et al., Predicting 30-Day Mortality for Patients With Acute Heart Failure in the Emergency Department A Cohort Study. Annals of Internal Medicine, 2017. 167(10): p. 698-+. |
| 67 | Senni, M., et al., Predicting heart failure outcome from cardiac and comorbid conditions: The 3C-HF score. International Journal of Cardiology, 2013. 163(2): p. 206-211. |
| 68 | Thorvaldsen, T., et al., Predicting Risk in Patients Hospitalized for Acute Decompensated Heart Failure and Preserved Ejection Fraction The Atherosclerosis Risk in Communities Study Heart Failure Community Surveillance. Circulation-Heart Failure, 2017. 10(12). |
| 69 | Mahajan, S.M., et al., Predicting Risk of 30-Day Readmissions Using Two Emerging Machine Learning Methods, in Nursing Informatics 2018: Ict To Improve Quality and Safety at the Point of Care, A.K. Rotegard, et al., Editors. 2018. p. 250-255. |
| 70 | Wang, L., et al., Predicting Risk of Hospitalization or Death Among Patients With Heart Failure in the Veterans Health Administration. American Journal of Cardiology, 2012. 110(9): p. 1342-1349. |
| 71 | Scrutinio, D., et al., Predicting Short-Term Mortality in Advanced Decompensated Heart Failure - Role of the Updated Acute Decompensated Heart Failure/N-Terminal Pro-B-Type Natriuretic Peptide Risk Score. Circulation Journal, 2015. 79(5): p. 1076-+. |
| 72 | Sartipy, U., et al., Predicting survival in heart failure: validation of the MAGGIC heart failure risk score in 51 043 patients from the Swedish Heart Failure Registry. European Journal of Heart Failure, 2014. 16(2): p. 173-179. |
| 73 | Au, A.G., et al., Predicting the risk of unplanned readmission or death within 30 days of discharge after a heart failure hospitalization. American Heart Journal, 2012. 164(3): p. 365-372. |
| 74 | Frizzell, J.D., et al., Prediction of 30-day all-cause readmissions in patients hospitalized for heart failure comparison of machine learning and other statistical approaches. JAMA Cardiology, 2017. 2(2): p. 204-209. |
| 75 | Wang, N., et al., Predictors of Frequent Readmissions in Patients With Heart Failure. Heart Lung and Circulation, 2019. 28(2): p. 277-283. |
| 76 | Cleland, J.G., et al., Predictors of Postdischarge Outcomes From Information Acquired Shortly After Admission for Acute Heart Failure A Report From the Placebo-Controlled Randomized Study of the Selective A1 Adenosine Receptor Antagonist Rolofylline for Patients Hospitalized With Acute Decompensated Heart Failure and Volume Overload to Assess Treatment Effect on Congestion and Renal Function (PROTECT) Study. Circulation-Heart Failure, 2014. 7(1): p. 76-87. |
| 77 | Simpson, J., et al., Prognostic Models Derived in PARADIGM-HF and Validated in ATMOSPHERE and the Swedish Heart Failure Registry to Predict Mortality and Morbidity in Chronic Heart Failure. Jama Cardiology, 2020. 5(4): p. 432-441. |
| 78 | Morbach, C., et al., Prognostic potential of midregional pro-adrenomedullin following decompensation for systolic heart failure: comparison with cardiac natriuretic peptides. European Journal of Heart Failure, 2017. 19(9): p. 1166-1175. |
| 79 | Cubbon, R.M., et al., Prospective development and validation of a model to predict heart failure hospitalisation. Heart, 2014. 100(12): p. 923-929. |
| 80 | Lee, D.S., et al., Prospective Validation of the Emergency Heart Failure Mortality Risk Grade for Acute Heart Failure The ACUTE Study. Circulation, 2019. 139(9): p. 1146-1156. |
| 81 | Ashfaq, A., et al., Readmission prediction using deep learning on electronic health records. Journal of Biomedical Informatics, 2019. 97. |
| 82 | Xiao, C., et al., Readmission prediction via deep contextual embedding of clinical concepts. Plos One, 2018. 13(4). |
| 83 | Jiang, W., et al., Readmission Risk Trajectories for Patients With Heart Failure Using a Dynamic Prediction Approach: Retrospective Study. JMIR medical informatics, 2019. 7(4): p. e14756-e14756. |
| 84 | Wessler, B.S., et al., Regional Validation and Recalibration of Clinical Predictive Models for Patients With Acute Heart Failure. Journal of the American Heart Association, 2017. 6(11). |
| 85 | Stampehl, M., et al., Risk assessment of post-discharge mortality among recently hospitalized Medicare heart failure patients with reduced or preserved ejection fraction. Current Medical Research and Opinion, 2020. 36(2): p. 179-188. |
| 86 | Andersson, C., et al., A risk score for predicting 30-day mortality in heart failure patients undergoing non-cardiac surgery. European Journal of Heart Failure, 2014. 16(12): p. 1310-1316. |
| 87 | Leong, K.T.G., et al., Risk Stratification Model for 30-Day Heart Failure Readmission in a Multiethnic South East Asian Community. American Journal of Cardiology, 2017. 119(9): p. 1428-1432. |
| 88 | Novack, V., et al., Routine Laboratory Results and Thirty Day and One-Year Mortality Risk Following Hospitalization with Acute Decompensated Heart Failure. Plos One, 2010. 5(8). |
| 89 | Clemens, M., et al., The Seattle Heart Failure Model Predicts Survival in Patients With Cardiac Resynchronization Therapy: A Validation Study. Journal of Cardiac Failure, 2012. 18(9): p. 682-687. |
| 90 | Karauzum, K., et al., A simple discharge risk model for predicting 1-year mortality in hospitalised acute decompansated heart failure patients with reduced ejection fraction. Acta Cardiologica, 2018. 73(2): p. 164-170. |
| 91 | Yap, J., et al., The Singapore Heart Failure Risk Score: Prediction of Survival in Southeast Asian Patients. Annals Academy of Medicine Singapore, 2019. 48(3): p. 86-94. |
| 92 | Felker, G.M., et al., Soluble ST2 in Ambulatory Patients With Heart Failure Association With Functional Capacity and Long-Term Outcomes. Circulation-Heart Failure, 2013. 6(6): p. 1172-1179. |
| 93 | O'Connor, C.M., et al., Triage After Hospitalization With Advanced Heart Failure The ESCAPE (Evaluation Study of Congestive Heart Failure and Pulmonary Artery Catheterization Effectiveness) Risk Model and Discharge Score. Journal of the American College of Cardiology, 2010. 55(9): p. 872-878. |
| 94 | Nakazone, M.A., et al., The use of the CALL Risk Score for predicting mortality in Brazilian heart failure patients. Esc Heart Failure, 2020. |
| 95 | Miro, O., et al., The Usefulness of the MEESSI Score for Risk Stratification of Patients With Acute Heart Failure at the Emergency Department. Revista Espanola De Cardiologia, 2019. 72(3): p. 198-207. |
| 96 | Mahajan, S.M. and R. Ghani, Using Ensemble Machine Learning Methods for Predicting Risk of Readmission for Heart Failure. Studies in health technology and informatics, 2019. 264: p. 243-247. |
| 97 | Montero-Perez-Barquero, M., et al., Utility of the SENIORS elderly heart failure riskmodel applied to the RICA registry of acute heart failure. International Journal of Cardiology, 2015. 182: p. 449-453. |
| 98 | Mahajan, S.M., et al., A Validated Risk Model for 30-Day Readmission for Heart Failure, in Medinfo 2017: Precision Healthcare through Informatics, A.V. Gundlapalli, M.C. Jaulent, and D. Zhao, Editors. 2017. p. 506-510. |
| 99 | Shiraishi, Y., et al., Validation and Recalibration of Seattle Heart Failure Model in Japanese Acute Heart Failure Patients. Journal of Cardiac Failure, 2019. 25(7): p. 561-567. |
| 100 | Quan, H., et al., Validation of Predictive Score of 30-Day Hospital Readmission or Death in Patients With Heart Failure. American Journal of Cardiology, 2018. 121(3): p. 322-329. |
| 101 | Regoli, F., et al., Validation of Seattle Heart Failure Model for mortality risk prediction in patients treated with cardiac resynchronization therapy. European Journal of Heart Failure, 2013. 15(2): p. 211-220. |
| 102 | Khanam, S.S., et al., Validation of the MAGGIC (Meta-Analysis Global Group in Chronic Heart Failure) heart failure risk score and the effect of adding natriuretic peptide for predicting mortality after discharge in hospitalized patients with heart failure. Plos One, 2018. 13(11). |
| 103 | Sudhakar, S., et al., Validation of the Readmission Risk Score in Heart Failure Patients at a Tertiary Hospital. Journal of Cardiac Failure, 2015. 21(11): p. 885-891. |
| 104 | Nagai, T., et al., Validation of US mortality prediction models for hospitalized heart failure in the United Kingdom and Japan. European Journal of Heart Failure, 2018. 20(8): p. 1179-1190. |
| 105 | Li, S., et al., Validity of the Seattle Heart Failure Model after heart failure hospitalization. Esc Heart Failure, 2019. 6(3): p. 509-515. |
| 106 | Laszczynska, O., et al., Validity of the Seattle Heart Failure Model for prognosis in a population at low coronary heart disease risk. Journal of Cardiovascular Medicine, 2016. 17(9): p. 653-658. |
| 107 | Falletta, C., et al., Additive Value of Biomarkers and Echocardiography to Stratify the Risk of Death in Heart Failure Patients with Reduced Ejection Fraction. Cardiology Research and Practice, 2019. 2019. |
| 108 | Nunez, J., et al., Antigen carbohydrate 125 and brain natriuretic peptide serial measurements for risk stratification following an episode of acute heart failure. International Journal of Cardiology, 2012. 159(1): p. 21-28. |
| 109 | Chen, Yuntao, et al. "A heart failure phenotype stratified model for predicting 1-year mortality in patients admitted with acute heart failure: results from an individual participant data meta-analysis of four prospective European cohorts." BMC medicine 19.1 (2021): 1-11. |
| 110 | Li, Hao, et al. "A novel age-biomarker-clinical history prognostic index for heart failure with reduced left ventricular ejection fraction." Open Medicine 15.1 (2020): 644-653. |
| 111 | Siriopol, Dimitrie, et al. "Application of survival classification and regression tree analysis for identification of subgroups of risk in patients with heart failure and reduced left ventricular ejection fraction." The International Journal of Cardiovascular Imaging (2021): 1-9. |
| 112 | Averbuch, Tauben, et al. "Derivation and validation of a two‐variable index to predict 30‐day outcomes following heart failure hospitalization." ESC Heart Failure (2021). |
| 113 | Ju, Chengsheng, et al. "Derivation of an electronic frailty index for predicting short‐term mortality in heart failure: a machine learning approach." ESC Heart Failure (2021). |
| 114 | Elias, Adi, et al. "SOFA score and short-term mortality in acute decompensated heart failure." Scientific reports 10.1 (2020): 1-10. |
| 115 | Garg, Nidhi, et al. "Performance of Emergency Heart Failure Mortality Risk Grade in the Emergency Department." Western Journal of Emergency Medicine 22.3 (2021): 672. |
| 116 | Sun, Yuxi, et al. "Predictive value of H2FPEF score in patients with heart failure with preserved ejection fraction." ESC Heart Failure 8.2 (2021): 1244-1252. |
| 117 | Chang, Hao-Chih, et al. "Risk stratification in patients hospitalized for acute heart failure in Asian population." Journal of the Chinese Medical Association 83.6 (2020): 544-550. |
